# Writing Abilities in Primary Progressive Aphasia: A Scoping Literature Review

**DOI:** 10.3390/brainsci16040420

**Published:** 2026-04-17

**Authors:** Valentina Esposito, Francesca Conca, Gaia C. Santi, Stefano F. Cappa, Eleonora Catricalà

**Affiliations:** 1 Istituti Clinici Scientifici Maugeri IRCCS, Milan Institute, 20138 Milan, Italy; espositovalentina.nps@gmail.com; 2 ICoN Cognitive Neuroscience Center, Institute for Advanced Studies, IUSS, 27100 Pavia, Italy; francesca.conca@iusspavia.it (F.C.); stefano.cappa@iusspavia.it (S.F.C.); 3 Istituti Clinici Scientifici Maugeri IRCCS, Bari Institute, 70124 Bari, Italy; gaiachiara.santi@gmail.com; 4 IRCCS Istituto Auxologico, 20145 Milan, Italy

**Keywords:** writing abilities, primary progressive aphasia, language

## Abstract

**Highlights:**

**What are the main findings?**

- Writing assessment in PPA is highly heterogeneous, with overreliance on dictation tasks.
- Distinct dysgraphia profiles emerge across PPA variants, while tasks targeting lexico-semantic, morpho-syntactic, and discourse-level writing are underrepresented.

**What are the implications of the main findings?**

- Comprehensive, linguistically informed writing tasks are needed to capture the full spectrum of writing impairments in PPA.
- Standardized approaches to writing assessment could improve diagnostic accuracy and cross-linguistic comparability in clinical and research settings.

**Abstract:**

Background: Given the central role of writing and typing in contemporary communication, integrating writing assessments into clinical practice is crucial for improving the diagnosis and management of primary progressive aphasia (PPA). This scoping review summarizes evidence on writing abilities in PPA, examining task types, their strengths and limitations, the linguistic features of stimuli, and the influence of language differences. Methods: A literature search was conducted using the Google Scholar and PubMed databases. We included papers published in peer-reviewed journals and written in English that present data from at least one PPA subject and report a quantitative score relative to a writing task. Fifty-one studies were included (forty-seven behavioral; four with neuroimaging). Results: Overall, the literature is fragmented, with marked variability in task design and the control of psycholinguistic variables. Writing to dictation is the most frequently used task but fails to capture the full spectrum of writing impairments, whereas tasks tapping lexico-semantic, morpho-syntactic, and discourse-level abilities are rarely employed. At the syndromic description level, svPPA typically shows surface dysgraphia, nfvPPA presents phonological dysgraphia and agrammatic writing, and lvPPA displays mixed error profiles. Neuroimaging findings are highly heterogeneous. Conclusions: The review underscores the need for systematic, linguistically grounded approaches to writing assessments in PPA to enhance diagnostic precision and cross-linguistic comparability.

## 1. Introduction

Writing is a fundamental communication skill essential for everyday activities and represents a relatively recent development in human history, having emerged approximately five thousand years ago [1]. As a culturally acquired ability, writing typically necessitates formal instruction, sustained effort, and years of practice to master. Much of our understanding of written production and its neural underpinnings comes from studies of individuals with acquired writing disorders (agraphia or dysgraphia) following focal brain damage, most commonly due to stroke [2,3,4,5]. This body of work has strongly informed the assessment of writing impairments in neurodegenerative diseases.

More recently, neuroimaging research with healthy individuals has provided important additional contributions [1,6].

The production of written words relies on multiple interacting cognitive processes, described in various models that commonly distinguish between central and peripheral components, each comprising several fine-grained sub-processes [7,8,9]. Central, spelling-specific processes typically begin either from the transcoding of auditory input or from internally generated word meanings. These processes involve the selection of the correct orthographic form, which depends on access to semantic knowledge and to the stored orthographic lexicon—i.e., the repository of known word spellings—primarily engaging temporal regions, such as the fusiform gyrus and the inferior temporal cortex. Spelling unknown words or non-words, in contrast, requires phonology-to-orthography conversion processes, which use learned mappings between sounds and letters to generate plausible spellings for phonological strings. This process is supported by the left posterior superior temporal, inferior parietal, and frontal areas [1]. The assembled or retrieved orthographic form is subsequently maintained and processed in the graphemic buffer, a limited-capacity system that sustains letter identity and order until they are transmitted to peripheral processes for execution. This buffer has been associated with the superior and middle frontal gyri as well as with the premotor cortex [1,6]. Peripheral components for written output (e.g., handwriting, typing) involve the stages from letter-shape selection to motor execution, supported by the left superior frontal and superior parietal cortex [1].

Different tasks can be employed to evaluate writing performance, and analyzing the differences across tasks can pinpoint the specific components that are affected. The most used one in the literature is oral dictation (of regular and irregular words, non-words, etc.). Specific patterns of quantitative and qualitative scores may identify impairments in particular components/processes, which are associated with distinct dysgraphia syndromes. The existence of the model’s components was verified and inspired by different dysgraphic patterns reported in focal neurological patients [10,11,12].

Central dysgraphias include the surface dysgraphia, marked by difficulties in spelling irregular words due to disruption of the stored orthographic lexicon, phonological dysgraphia, due to phoneme-to-grapheme conversion impairment, leading to an inability to spell non-words, and graphemic buffer agraphia, characterized by letter omissions, substitutions, additions, and transpositions. Multiple forms of peripheral dysgraphia affecting allographic and graphomotor stages have also been reported [13].

Beyond single-word assessment, a writing evaluation can include connected written production, allowing for the evaluation of written morphosyntactic abilities. This is particularly valuable given that syntactic and grammatical deficits are central in many neurological conditions, especially in Primary Progressive Aphasia (PPA), and may manifest not only in speech but also in writing, thereby offering additional and distinct evidence of underlying language dysfunction.

Patients with PPA present selective language deficits that often extend in relatively specific ways to writing [14]. In the semantic variant of PPA (svPPA), characterized by semantic memory deficits linked to anterior temporal lobe involvement, surface dyslexia with difficulties in spelling irregular words emerges as a diagnostic feature [15,16]. In the non-fluent PPA variant (nfvPPA), characterized by agrammatism and/or speech apraxia due to inferior frontal lobe damage, phonological dysgraphia is observed, with difficulties in writing non-words, often associated with reduced syntactic complexity and morphological errors [12,17]. In logopenic PPA (lvPPA), characterized by deficits in lexical retrieval and phonological short-term memory associated with the involvement of the parieto-temporal junction, writing impairments are heterogeneous, showing features of both surface and phonological dysgraphia [12]. Mixed PPA cases, exhibiting overlapping or partial variant features [18,19], are associated with writing difficulties in 40–75% of patients [20], typically affecting spelling abilities [21].

Despite the relevance of writing difficulties in PPA, only a limited number of studies have been conducted. Additionally, the available investigations are heterogeneous, with most research focusing on spelling through dictation [22,23,24]. They also differ in the psycholinguistic features of the stimuli, which are known to influence writing performance [9,13,25,26,27,28]. Another crucial factor is the language-specific writing system, since different orthographies place distinct demands, as in the case of alphabetic versus logographic and ideographic scripts. Even within each system, different patterns may emerge. For example, phoneme-to-grapheme conversion is more challenging in opaque languages (e.g., English) than in transparent ones (e.g., Italian) [29,30].

This scoping review aims to map and synthesize the available evidence on written production in individuals with primary progressive aphasia (PPA), considering the range of tasks used for assessment, their strengths and limitations, the linguistic characteristics of the stimuli, and the influence of language differences on performance. Given the substantial heterogeneity in writing tasks and outcome measures across studies, a scoping review was considered the most appropriate approach to map the breadth and variability of the existing literature. Unlike systematic reviews, which aim to answer specific and narrowly defined questions, scoping reviews allow for a broader mapping of the literature and the identification of key concepts and gaps.

Unlike previous reviews, which did not specifically focus on PPA, e.g., including different types of dementia [16], or focused on a single writing task, e.g., spelling [12], or on a single language (e.g., Chinese) [31], the present scoping review offers a broader perspective while specifically focusing on PPA. In line with the Joanna Briggs Institute methodological guidance, the scope of the review was defined according to the Population–Concept–Context (PCC) framework [32] as follows:

- Population: Individuals with primary progressive aphasia (all variants).
- Concept: Writing abilities, including task types, performance, and error profiles.
- Context: Experimental and clinical studies assessing written language across different languages and orthographic systems.

## 2. Materials and Methods

The scope of the review and the inclusion criteria were defined according to the Population–Concept–Context (PCC) framework, as recommended by the Joanna Briggs Institute (JBI) for scoping reviews [32]. A comprehensive literature search was conducted using the Google Scholar and PubMed databases by two authors (V.E., G.C.S.). No formal review protocol was preregistered (e.g., on OSF or other repositories).

**Figure 1:**
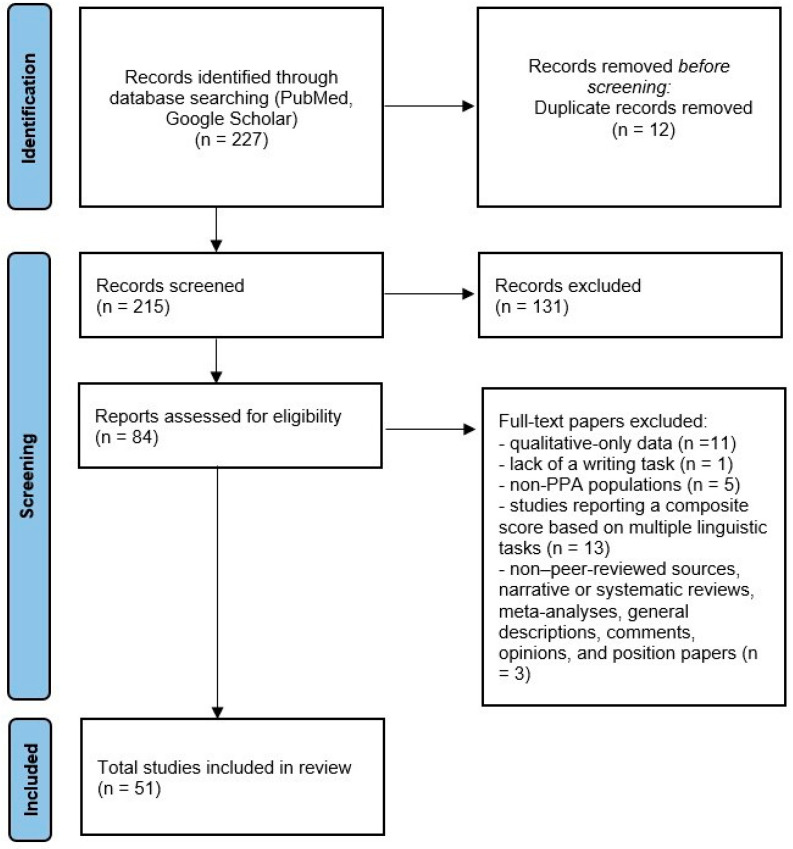
PRISMA-ScR flow diagram of the study selection process. The diagram reports the number of records identified, screened, assessed for eligibility, and included in the review.

The study selection process is illustrated in Figure 1 and follows the PRISMA guidelines for scoping review [33]; see the Supplementary Materials for the checklist.

The selection of databases was guided by the interdisciplinary nature of the topic. PubMed was chosen for its coverage of biomedical literature, while Google Scholar was included to capture a broader range of studies across clinical, neuropsychological, and linguistic domains. The review procedures were defined a priori and applied consistently throughout the study. To ensure comprehensive coverage, we used multiple combinations of keywords referring to Primary Progressive Aphasia and its variants, using different Boolean operators to ensure comprehensive coverage of behavioral and neuroimaging studies on writing in PPA (primary progressive aphasia OR semantic variant of Primary Progressive Aphasia OR logopenic variant of Primary Progressive Aphasia OR non-fluent variant of Primary Progressive Aphasia OR unclassifiable variant of Primary Progressive Aphasia), AND writing-related constructs (writing OR writing skills OR writing abilities OR writing impairment OR writing assessment) AND dysgraphia subtypes (dysgraphia OR surface dysgraphia OR deep dysgraphia) AND neuroimaging modalities (Magnetic Resonance Imaging OR functional Magnetic Resonance Imaging OR Voxel Brain Morphometry OR Positron Emission Tomography). The search covered all available records from database inception to November 2025. To ascertain the inclusion of all the relevant literature, a further manual search was conducted involving papers cited or citing previous reviews or descriptive studies (i.e., [12,22,23]), as well as from the lists of references of the retrieved papers.

The bibliographic search led to a total of 227 papers.

To be included, papers had to (1) appear in peer-reviewed journals and be written in English; (2) present data from at least one PPA subject, either with or without a specific variant diagnosis; and (3) report a quantitative score relative to a writing task, in order to ensure methodological comparability across studies and enable a structured synthesis of findings across different tasks, PPA variants, and languages. We included studies reporting results with a comparison with a healthy control group (HC) and/or with normative data and/or reporting results with a comparison among PPA variants and/or with one or more other clinical condition(s) (e.g., Mild Cognitive Impairment (MCI); Alzheimer’s disease (AD), Cortico-Basal Degeneration (CBD); Progressive Supranuclear Palsy–Richardson syndrome (PSP-RS)). Studies reporting brain imaging data (structural or functional) with Regions of Interest (ROIs) and/or whole-brain analysis related to written language skills were also included.

Studies were excluded when no writing task was administered. We also excluded studies involving populations other than individuals with Primary Progressive Aphasia, as well as those in which PPA-specific data could not be isolated from mixed samples. Publications not written in English were not eligible, to ensure accurate screening and interpretation of the retrieved studies. Studies reporting only composite language scores derived from multiple linguistic tasks, without writing-specific measures, were also excluded. Finally, non-peer-reviewed sources, along with publication types not eligible for inclusion—such as narrative or systematic reviews, meta-analyses, general descriptive papers, commentaries, opinion pieces, and position papers—were not considered.

Studies reporting exclusively qualitative data were not included, as the absence of quantitative measures would have limited comparability across studies and hindered the possibility of systematically synthesizing findings within a unified framework. This choice was made to reduce conceptual variability and enhance the interpretability of the mapped evidence, given the high heterogeneity of tasks, scoring procedures, and reporting standards in the literature.

For longitudinal studies, all eligible time points were included and treated as separate descriptive instances, integrated within a narrative synthesis, rather than as statistically independent observations. In treatment studies, only baseline data were considered to ensure comparability.

To increase consistency, both authors (V.E., G.C.S.) independently screened all retrieved publications and assessed their eligibility for inclusion. Disagreements occurred in a limited proportion of cases (5 out of 227 records) and were resolved through discussion and consensus, with the involvement of a third author (E.C.) when necessary. A structured data charting form was developed a priori to extract relevant information from each included study, in line with the JBI methodological guidance for scoping reviews. The extracted information included: (1) study characteristics (e.g., type of task, language, number and type of patients, main results); (2) detailed description of writing tasks (including stimulus properties, task instructions, and scoring procedures); (3) qualitative error definitions; and (4) linguistic features derived from written description tasks.

Data extraction was performed independently by two authors (V.E., G.C.S.). Any discrepancies were resolved through discussion and consensus, with the involvement of additional authors when necessary. In line with JBI guidance, the charting process was iterative, allowing for the refinement of data categorization during the review process [32].

A total of 51 studies fulfilled the selection criteria and were included in the review.

## 3. Results

Out of 51 reviewed studies, 47 included only neuropsychological data, whereas 4 additionally reported neuroimaging data, i.e., Magnetic Resonance Imaging (MRI) in all cases. Twenty-six were group studies and twenty-five were single case reports. Nine of the included papers reported longitudinal data. Most of the studies were conducted in English (59%), followed by French (11%), Spanish (10%), Italian (6%), Japanese (6%), Polish (4%), and other languages, e.g., German and Chinese (2%).

The clinical conditions included in the reviewed papers are reported in Table 1, together with the number of studies investigating each of them. Non-classifiable and mixed PPA were not considered as a specific target condition because of the heterogeneity of their clinical presentation.

The 51 reviewed studies presented behavioral scores obtained from five writing tasks; see Table 2 for a description. The classification into five categories was derived inductively from the included studies. When studies included multiple writing components, each component was considered separately and classified according to its specific characteristics, such as the type of linguistic material (e.g., words, nonwords, sentences) within the same task.

**Figure 2:**
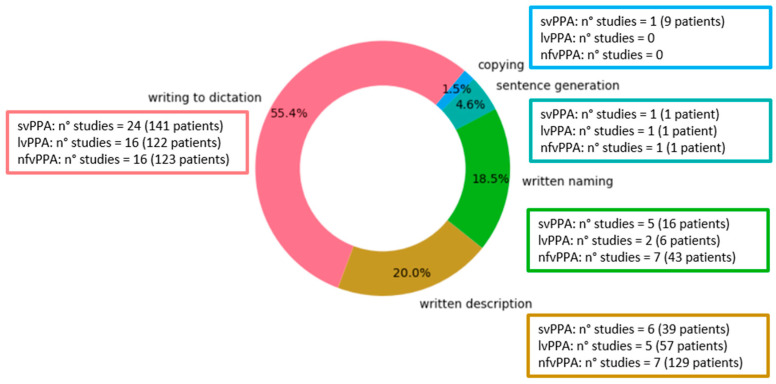
Frequency of use of the five writing tasks in the included studies. The figure illustrates the relative frequency with which each writing task was employed across the included studies. The categorization of writing tasks into five groups was derived inductively from the tasks reported in the included papers. All tasks reported in the included studies were considered in the analysis. This representation supports interpretation by providing an overview of the task distribution across the literature and by indicating areas where findings may be limited due to the underrepresentation of specific tasks. For each task, we additionally report the number of studies, including each PPA variant (svPPA, lvPPA, nfvPPA) and the total number of patients, calculated by summing the sample sizes across all contributing studies. Notably, studies including more than one PPA variant were counted separately for each variant (e.g., a study including both svPPA and lvPPA contributes to both categories). Longitudinal studies were counted only once. This representation allows for assessment of whether different writing tasks are unevenly represented across PPA variants, helping to identify variants for which specific tasks may be underrepresented. Accordingly, findings related to underrepresented task–variant combinations should be interpreted with caution.

The frequency of task usage varied across studies, with writing to dictation being the most employed task, while copying was the least utilized, appearing in only one study (see Figure 2).

In addition to overall performance, error types were analyzed across the five tasks. The classification and definitions of errors varied between studies, often depending on the specific language under investigation. Error definitions were systematically compared only within the written-to-dictation tasks, which represented the most consistently reported condition across studies. In this subset, we grouped error types into three broad categories—phonologically plausible, non-phonologically plausible, and lexico-semantic—when definitions and/or examples clearly referred to the same underlying phenomenon, despite differences in terminology. In all other cases, no merging was performed, as error types were not fully overlapping or reflected task- or language-specific characteristics.

Furthermore, several studies considered the influence of stimulus characteristics and/or psycholinguistic variables on writing performance, and specifically the effect of the frequency of stimuli (*n* = 9 studies), length (*n* = 4 studies), imageability (*n* = 3 studies), and concreteness and familiarity (*n* = 2 studies).

In the Supplementary Materials we reported additional information regarding the included studies, as follows: (1) the detailed results for each study (Supplementary Table S1); (2) the characteristics of each reviewed task in terms of language, stimuli, psycholinguistic features, and scoring (Supplementary Table S2); (3) the qualitative errors reported in written to dictation in each study alongside the respective definition (Supplementary Table S3); (4) the features considered in the written description and written sentence generation alongside the definition used in each study (Supplementary Table S4).

### 3.1. Behavioral Results

For each study we took into account the results obtained from the comparison among the PPA variants, or between PPA and the group of healthy controls, or between PPA and the other clinical conditions (as shown in Table 1). The main behavioral results are summarized in Table 3, showing only effects and comparisons assessed by at least two studies. For a detailed presentation of all the findings, see the Supplementary Materials and Supplementary Table S1.

As reported in Table 3, performance on real words in writing-to-dictation tasks (i.e., without distinguishing between regular and irregular words) is generally impaired in svPPA and lvPPA. In contrast, nfvPPA shows a performance comparable to healthy controls. Longitudinal data indicate a decline in svPPA.

Error patterns differ across variants, with svPPA producing more phonologically plausible errors (PPEs), nfvPPA producing more phonologically implausible errors (non-PPEs), and lvPPA showing no difference between the two error types. The influence of variables is little investigated; available findings indicate effects of frequency and regularity in svPPA, and no effect of word length in nfvPPA. Writing to dictation of regular words tends to be preserved except in lvPPA, whereas deficits in irregular words are reported in both lvPPA and svPPA, with svPPA showing the greatest impairment. Writing to dictation of non-words is particularly impaired in lvPPA, while it is preserved in svPPA relative to the writing to dictation of words. Written naming is largely preserved in nfvPPA—apart from object naming—and remains stable over time. In written description tasks, nfvPPA shows a reduced number of information units and an impoverished written output, characterized by fewer words, verbs, function words, content words, and words per minute compared to healthy controls. svPPA produces fewer information units and shows impaired performance on a global score, while lvPPA does not differ from healthy controls on the same global measure. Notably, copying and written-sentence generation have been examined in only a few studies, and no consistent patterns have emerged.

In summary, across tasks, writing impairments in PPA show variant-specific profiles. svPPA typically presents with surface dysgraphia in writing-to-dictation tasks and reduced information content in written description tasks. NfvPPA is characterized by phonological dysgraphia in writing to dictation and agrammatism in written description. LvPPA, in contrast, does not display a consistent profile.

Evidence regarding task variables, as well as less-studied abilities (e.g., copying, written sentence generation) and features (e.g., within written description tasks), remains limited and highly heterogeneous, highlighting the need for more systematic investigations.

### 3.2. Brain Imaging Results

Two different tasks have been used in neuroimaging studies, namely writing to dictation and written description.

Three studies explored the neural correlates of writing-to-dictation tasks. They varied for the type of stimuli, including real, regular, irregular, and/or non-words, and the number of stimuli, ranging from 10 (PALPA) [50] to 326 (Johns Hopkins Dysgraphia Battery) [51].

Two studies correlated brain imaging data with the performance of all PPA, considering accuracy and error type, using either whole-brain- [50], or ROIs-based analysis [35].

Accuracy for non-words positively correlated with the volume of the left superior/inferior parietal lobe, the left precentral, and the postcentral gyrus [35]. Spelling errors produced on non-words negatively correlated with the cortical thickness in the left inferior frontal gyrus (IFG), pars orbitalis, and the left supramarginal gyrus, while spelling errors on irregular words negatively correlated with the cortical thickness in the left temporal pole and fusiform gyrus. NonPPEs negatively correlated with the cortical thickness in the left IFG, encompassing both the pars opercularis and the pars triangularis [50].

One study explored the cortical thickness of four different profiles identified across PPA variants, considering both accuracy and error type [51]. Only one pattern, characterized by surface dysgraphia, with a deficit in spelling of irregular words, and the production of PPEs negatively correlated with the cortical thickness in the anterior temporal lobe [51]. Further patterns are reported in the Supplementary Materials.

Overall, writing-to-dictation performance in PPA appears to rely on partially dissociable neural networks depending on lexicality. Non-word spelling errors are primarily associated with left fronto-parietal regions, consistent with phonological and grapheme–phoneme conversion mechanisms. In contrast, irregular word spelling deficits and surface dysgraphia are linked to anterior temporal regions, supporting the lexical–semantic route.

One study correlated the features derived from a written description task with the gray matter volume [72]. The volume in Broca’s area negatively correlated with the production of ungrammatical sentences and semantic errors in the whole PPA group, and positively correlated with the proportion of correctly produced verbs when restricting the analysis to nfvPPA and nfv+POAS (primary progressive apraxia of speech) patients.

**Figure 3:**
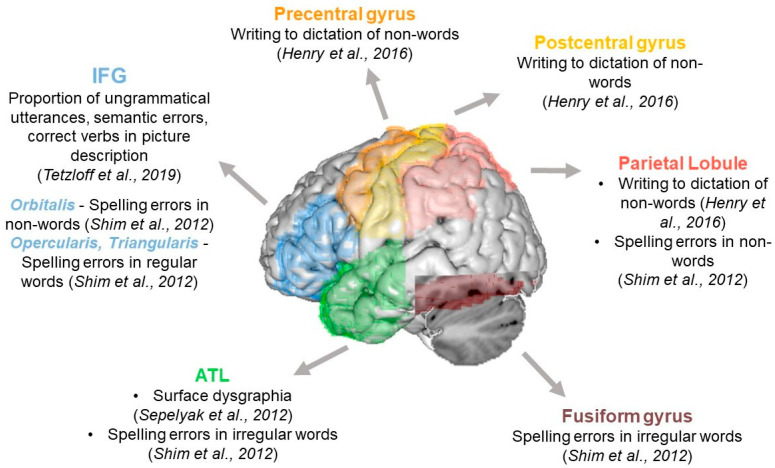
3D anatomical rendering of the brain regions involved in the five writing tasks. The figure displays the brain regions associated with performance in writing tasks, as reported across the included studies. Regions were identified according to the anatomical labels provided in each paper and mapped onto the Automated Anatomical Labeling (AAL) atlas [73]. For the anterior temporal lobe (ATL), a single composite region is shown, obtained by combining the portions anterior to Y = −58 (inferior temporal gyrus), −56 (middle temporal gyrus), and −51 (superior temporal gyrus), together with the temporal pole, following the anterior/posterior partitions [74]. Broca’s area, as reported [72], is represented as the inferior frontal gyrus (IFG) in accordance with AAL conventions. This approach ensures consistency in anatomical labeling across studies and supports interpretation by clarifying how heterogeneous regional definitions were harmonized for synthesis. Abbreviations: IFG = inferior frontal gyrus; ATL = anterior temporal lobe [35,50].

For a summary of the imaging results, see Figure 3 and Supplementary Materials for additional information.

The available data, although limited, suggest that frontoparietal regions are associated with sublexical processes, anterior temporal regions underpin lexical–semantic functions, and inferior frontal cortices contribute to syntactic and lexico-semantic processing during extended written production. This latter interpretation is supported by the involvement of these regions in the production of ungrammatical sentences, semantic errors, and verb-related impairments.

However, the available neuroimaging studies are few and highly heterogeneous, and the findings should therefore be interpreted with caution.

## 4. Discussion

This review offers an overview of the writing tasks used in PPA, alongside their linguistic features, showing a limited and fragmented body of evidence, characterized by considerable variability in tasks, measures, and stimulus variables.

### 4.1. Writing Tasks

A variety of tasks have been employed in the literature, each assessing distinct language domains and potentially helping to delineate the linguistic patterns of different PPA variants. In addition to the most widely used task, writing to dictation—present in more than 50% of the reviewed studies and useful for detecting different dysgraphia patterns—other tests capture additional relevant aspects of written language.

Written naming tasks, for instance, are needed for probing the retrieval of lexico-semantic and orthographic information from long-term memory. Written sentence generation can provide noteworthy information about patients’ morpho-syntactic competence, especially when stimuli vary in length and syntactic complexity, thus posing distinct challenges to logopenic and non-fluent variants. Connected writing tasks, in turn, offer a more ecological perspective on morpho-syntactic and discourse abilities, allowing for the simultaneous analysis of multiple linguistic dimensions—ranging from informativeness to semantics and syntax—all differentially affected across PPA variants. These tasks, however, are still rarely used in clinical settings, mainly because they are time-consuming and difficult to score—similarly to what has been reported for oral assessments, which, although more widely employed, present comparable challenges [75]. Further variability across studies arises from differences in, or even the omission of, consideration of key psycholinguistic variables such as word frequency and familiarity, which are known to strongly influence performance, further limiting the comparability and interpretability of results. Additionally, inconsistencies in how writing errors are defined contribute to the low generalizability of findings.

Overall, this heterogeneity partly reflects the inadequacy of the theoretical frameworks typically guiding writing assessment, mainly derived from post-stroke aphasia [2,3,4,5].

These frameworks fail to fully capture the dynamic and progressive nature of neurodegenerative language disorders, which are characterized by distinct lesion sites and trajectories [76].

### 4.2. Written Profile of PPA Variants

Overall, the emerging pattern—although derived from very few studies—appears consistent with the core features of each variant, while also revealing additional, clinically relevant information that should not be overlooked and needs further exploration.

#### 4.2.1. Semantic Variant of PPA

Across the reviewed studies, the writing profile observed in svPPA appears consistent with the core and secondary features of the syndrome, namely deficits in semantic memory and the presence of surface dysgraphia, respectively.

In written picture description, svPPA patients showed a reduced production of information units, aligning with evidence from connected speech, typically characterized by a semantically impoverished output [77].

The presence of surface dysgraphia was clearly reported in studies adopting the writing-to-dictation task, showing a deficit in irregular words (e.g., knife, yacht) compared to healthy subjects and to the other PPA variants [52,53,54,56,57,60]. The typical phonologically plausible errors (PPEs) (e.g., yacht as yot) are identifiable across languages, including English, Italian, Chinese and Japanese [34,36,48,50,51,78], and have been proposed as a distinctive feature of svPPA compared to other variants [23].

In addition, svPPA patients reported a spared performance for non-words [35,40,44,53,57,63,64] and sentences [38,44], and a higher accuracy for high- than for low-frequency words [34,36,52,53].

Performance on real words decreased over time [34,39], a pattern attributed to the disruption of the stored orthographic lexicon in svPPA [12], due to the left temporal regions’ involvement [51].

Overall, the writing pattern can be attributed to the loss of conceptual knowledge and the progressive general language impairment experienced by individuals with svPPA [75,79,80,81,82].

#### 4.2.2. Non Fluent Variant of PPA

Writing tasks in nfvPPA appear to be effective in detecting one of the core diagnostic features, i.e., agrammatism. In particular, connected writing revealed short and syntactically simplified productions, namely characterized by a reduced number of words, words per minute, verbs, content and function words [17,24,69]. This pattern parallels the findings from connected speech, where patients showed a diminished speech rate and an impaired ability to produce complex syntactic structures [75], specifically producing a low number of words per utterances and displaying syntactic and inflectional errors [17,83,84,85]. Additionally, the reduction in informativeness, in terms of the number of written information units [17,24,69], was also reported in connected speech [75].

The presence of phonological dysgraphia was reported in studies using writing to dictation, with more difficulties in spelling non-words when compared to svPPA [44,48,49,50]—although not showing differences when compared to healthy controls [35,44]. Performance on spelling real words was comparable to that of healthy individuals [35,44,45,46,47], and no effect of word length was reported [46,55]. Spelling errors mostly included phonologically implausible strings (nonPPEs), usually carrying some visual or orthographical similarity with the target word (e.g., summer as sumree) [50,51]. The production of nonPPEs has been associated with lesions affecting the left perysilvian territory, in particular the inferior frontal and precentral gyri, and the insula [28], areas known to be involved in nfvPPA [15].

Written naming tasks yielded mixed findings. Three single cases showed preserved performance when considering overall accuracy [47,65], while two group studies reported impaired performance when analyzing only object naming [17,44]. Such discrepancies may reflect differences in disease severity—for example, the language impairment was only mild in Thompson et al. 2007 [47], but more pronounced in Graham et al. 2004 [17]. Additionally, most of the studies reported a stable performance over time [66,67]. Overall, these findings are consistent with observations from oral picture naming, which also indicate a possible deficit [86] that is generally less severe than in other PPA variants [35].

#### 4.2.3. Logopenic Variant of PPA

The reviewed studies identified a heterogeneous writing profile in lvPPA, consistent with the general language profile of this variant [87].

Limited evidence is available from written description tasks, with only a preserved global score consistently reported across studies [59,71]. In contrast, writing-to-dictation tasks revealed a more complex pattern of impairment. While the performance was impaired in most of the reviewed studies, no clear specificity for the type of stimuli was observed. LvPPA indeed performed worse than healthy controls in regular and irregular words [43,58], as well as in non-words [35,40,42,44,58,63]. Additionally, the performance for non-words was worse than words in comparison to either nfvPPA [35,44] and svPPA [35,44,48]. Non-words are often misspelled and lexicalized, plausibly reflecting an attempt to compensate for phonological difficulties by relying on relatively spared semantic and orthographic knowledge [12]. Notably, in regular and irregular words spelling, lexical retrieval deficit might reduce the possibility of accessing the correct letter-sequence, thereby suggesting difficulties at both the sub-lexical and lexical levels [12,51]. Qualitatively, lvPPA produced both PPEs and nonPPEs [50,51].

The pattern of impairment, predominantly observed in dictation-based writing tasks, further supports the hypothesis that in the logopenic variant Primary Progressive Aphasia (lvPPA), pure phonological or semantic deficits are uncommon. Instead, spelling difficulties tend to reflect a hybrid profile, involving both phonological and semantic processing components [12].

### 4.3. Conclusions

The reviewed studies highlight the clinical and research relevance of writing assessment in PPA, as different tasks hold strong potential for revealing variant-specific linguistic deficits. Although the available evidence remains limited and fragmented—particularly compared with research on oral language—writing can be especially valuable for patients with severe speech impairments, such as those with nfvPPA. In these individuals, oral production may be markedly effortful or unintelligible [17].

Our review indicates that the use of writing tasks is not uniform across the literature. Some tasks are relatively infrequently employed, which may limit the robustness of the conclusions drawn for those specific tasks. Differences were also observed in the distribution of studies and number of patients across tasks, suggesting that findings related to underrepresented task–variant combinations should be interpreted with caution.

Writing-to-dictation tasks were the most frequently used, despite assessing a limited range of linguistic domains. They were more commonly employed in studies focusing on svPPA, as they are particularly suited to detecting surface dysgraphia, a supportive feature of this variant.

The typical phonologically plausible errors were observed across languages, including English, Italian, and Chinese [34,36,48,50,51]. However, the diagnostic sensitivity of this pattern varies across orthographic systems. While this pattern is prominent in opaque languages like English due to unpredictable phoneme–grapheme correspondences, it is less evident in transparent languages such as Italian, where there is high consistency between orthography and phonology. As a result, reliance on this marker alone may lead to the under-detection of svPPA in non-English-speaking populations. Accordingly, alternative markers [88,89], such as the inflection of irregular verbs [90], have been proposed to detect similar underlying semantic deficits. In addition, language-specific features, such as visual errors in logographic systems, may provide complementary diagnostic information.

Written description tasks, which allow for the assessment of multiple linguistic domains simultaneously, including discourse and syntactic processing, represent the second most frequently used approach. These tasks are used particularly frequently in non-fluent patients and have consistently enabled the identification of agrammatism, a core diagnostic feature of this variant.

Imaging studies are very limited but suggest that frontoparietal regions are associated with sublexical processes, anterior temporal regions underpin lexical–semantic functions, and inferior frontal cortices contribute to syntactic and lexico-semantic processing. However, variability across studies, driven by multiple sources of heterogeneity—including differences in writing tasks, patient samples, and disease severity, as well as distinct neuroimaging analytical approaches—precludes consistent conclusions and warrants cautious interpretation.

Some limitations should be acknowledged. To ensure a minimum level of methodological comparability, we excluded studies reporting only qualitative data, which may have limited the inclusion of potentially relevant evidence. In addition, as the literature search was conducted using PubMed and Google Scholar to allow for broad interdisciplinary coverage, the inclusion of additional indexed databases may have further increased the comprehensiveness and reproducibility of the review.

A further limitation concerns the restriction to English-language publications. Although this choice was made to ensure accurate screening and interpretation of the studies, it may have introduced a language bias and limited the inclusion of relevant evidence published in other languages. This is particularly relevant given the cross-linguistic variability of writing systems, which represents a central aspect of the present review. In addition, writing systems may involve other cognitive domains, such as attention and visuospatial skills, which may vary across languages and cultures [91].

In conclusion, writing assessment may represent a promising and accessible approach to refining PPA characterization and deepening our understanding of language functioning in both health and disease. This is particularly relevant given the central role of writing and typing in contemporary communication, especially among digitally active individuals.

While not intended as formal clinical recommendations, the available evidence suggests that combining writing-to-dictation and written description tasks may provide a more comprehensive assessment of written production in PPA, as these tasks capture complementary linguistic processes. However, the assessment of writing skills should be further developed and more widely implemented before a fully informative and reliable clinical picture can be established.

## Figures and Tables

**Table 1:** Clinical conditions included in the review. This table reports all clinical conditions represented in the included studies, together with their abbreviations and the number of studies addressing each condition. Conditions were grouped into target PPA variants and other neurological conditions according to predefined eligibility criteria. This structure supports interpretation, making it possible to identify conditions that are over- or under-represented.

**Target Clinical Conditions**
Semantic variant of Primary Progressive Aphasia (svPPA) (*n* = 30); Non-fluent variant of Primary Progressive Aphasia (nfvPPA) (*n* = 25); Logopenic variant of Primary Progressive Aphasia (lvPPA) (*n* = 21); PPA diagnosis, without a variant specification (*n* = 3).
**Other Clinical Conditions**
Alzheimer’s disease (AD) (*n* = 3); Apraxia of Speech (AOS) (*n* = 2); Cortico-Basal Degeneration (CBD) (*n* = 1); Mild Cognitive Impairment (MCI) (*n* = 1); Progressive Supranuclear Palsy–Richardson syndrome (PSP-RS) (*n* = 1).

**Table 2:** Description of the five written tasks, including their respective advantages and limitations.

Writing Task	Description	Advantages	Limitations
Copying	To copy a target, e.g. words, that is usually presented on a screen or paper for a limited amount of time	-Informative at orthographic, working memory, and graphomotor levels -Easy to administer and score -Short task duration	-Not informative for informativeness, lexical-semantic, morpho-syntactic level
Writing to dictation	To write stimuli, e.g. words, non-words, or sentences, after they were read aloud by the experimenter	-Informative at lexical-semantic, sub-lexical-orthographic, morphological, working memory, and graphomotor levels -Sensitive in the case of opaque orthographies -May include different types of stimuli: regular words, irregular words, non-word, sentences -Easy to administer and score -Short task duration	-Not informative at informativeness and morpho-syntactic levels -Less informative in the case of transparent orthographies
Written naming	To write the name of a visually presented image	-Informative at lexical-semantic, orthographic, morphological, graphomotor, and visual levels -May include different types of stimuli: object, action, picture -Easy to administer and score -Short task duration	-Not informative at informativeness and morpho-syntactic levels -Lack of homogeneous qualitative scoring procedures
Sentence generation	To generate a sentence from a cue	-Informative at morpho-syntactic, lexical-semantic, orthographic, and graphomotor levels -Easy to administer and score -Short task duration	-Less informative for informativeness
Written description (written picture description and spontaneous writing)	To describe a complex picture or to write freely about a topic	-Informative at lexical-semantic, orthographic, graphomotor levels -Well-defined structure enabling comparison across subjects -Easy to administer -Informative at informativeness, and morpho-syntactic levels (mostly for spontaneous writing) -Short task duration (only for written picture description)	-Difficult to score -Less informative for informativeness and morpho-syntactic levels (for written picture description in comparison to spontaneous writing) -Subjects tend to write single words rather than full sentences (only for written picture description) -Time requirements (only for spontaneous writing) -The lack on a well-defined structure leads to difficulty in comparing across subjects (only for spontaneous writing)

**Table 3:** Summary of effects and comparisons reported in at least two studies across the writing tasks. In the table it is reported when the performance was equal (=), worse (<), or better (>) in the comparisons between PPA and HC and between PPA variants. Only effects assessed by at least two studies were retained, reflecting the thresholds applied during data synthesis. Results reported in more than 50% of the studies are presented as main findings; inconsistent results—defined as cases in which no single effect direction emerged in more than half of the studies—are indicated as incosistent. PPA = Primary Progressive Aphasia; svPPA = semantic variant of Primary Progressive Aphasia; nfvPPA = non fluent variant of Primary Progressive Aphasia; lvPPA = logopenic variant of Primary Progressive Aphasia; HC = healthy controls; PPEs = phonologically plausible errors; nonPPEs = phonologically implausible errors.

Tasks	Comparisons and Effects Explored in at Least 2 Studies	Main Results, i.e., Reported in More than 50% of the Cases
Copying	No	-
Writing to dictation	Yes	Real words svPPA < HC [34,35,36,37,38,39,40] lvPPA < HC [35,36,40,41,42,43,44] nfvPPA = HC [35,44,45,46,47] nfvPPA = lvPPA = svPPA [36,45,48,49] Longitudinal svPPA decline over time [34,39] nfvPPA: inconsistent findings [34,39] Errors svPPA: PPEs > nonPPEs [34,36,48,50,51] lvPPA: PPEs = nonPPEs [50,51] nfvPPA: nonPPEs > PPEs [50,51] Effects svPPA: frequency [34,36,52,53] svPPA: regularity [52,53,54] nfvPPA: no effect of length [46,55] nfvPPA: inconsistent effects of imageability and regularity [46,55] Regular words svPPA = HC [52,53,54,56,57] lvPPA < HC [43,58] nfvPPA = lvPPA = svPPA [50,59] Irregular words svPPA < HC [52,53,54,56,57,60] lvPPA < HC [43,58] svPPA < lvPPA; svPPA < nfvPPA [50,59] Non-words PPA = HC [61,62] svPPA = HC [34,35,40,44,53,57,63,64] lvPPA < HC [35,40,42,44,58,63] nfvPPA = HC [35,44] nfvPPA < svPPA [44,48,49,50] lvPPA < svPPA [49,50] lvPPA < nfvPPA [35,44] Effects svPPA non word > word [35,53] lvPPA non-word < word [35,42] Sentences svPPA = HC [38,44]
Written Naming	Yes	Overall performance nfvPPA = HC [47,65] Objects naming nfvPPA < HC [17,44] svPPA: inconsistent findings [38,44,56] Longitudinal nfvPPA: stable over time [66,67,68] Effects nfvPPA: inconsistent effects of word length [17,66]
Generation of written sentences	No	-
Written description	Yes	nfvPPA < HC: number of information units, words, verbs, function words, content words, words per minute [17,24,69] nfvPPA vs. HC: inconsistent findings for number of nouns [17,69] svPPA < HC: information unit [24,70] and global score [64,71] lvPPA = HC: global score [59,71]

## Data Availability

The original contributions presented in this study are included in the article/Supplementary Materials. Further inquiries can be directed to the corresponding author.

## Supplementary Materials

The following supporting information can be downloaded at: https://www.mdpi.com/article/10.3390/brainsci16040420/s1, PRISMA checklist; Table S1: Results; Table S2: Tasks; Table S3: Errors definition; Table S4: Features definition; Supplementary Materials_final, providing a comprehensive and in -depth description of all the results presented. References [92,93,94,95,96,97,98] were cited in Supplementary Materials.

## References

[1] Purcell J.J., Turkeltaub P.E., Eden G.F., Rapp B. (2011). Examining the central and peripheral processes of written word production through meta-analysis. Front. Psychol..

[2] Cloutman L., Gottesman R., Chaudhry P., Davis C., Kleinman J.T., Pawlak M., Herskovits E.H., Kannan V., Lee A., Newhart M. (2009). Where (in the brain) do semantic errors come from?. Cortex.

[3] Lubrano V., Roux F.-E., Démonet J.-F. (2004). Writing-specific sites in frontal areas: A cortical stimulation study. J. Neurosurg..

[4] Sakurai Y., Onuma Y., Nakazawa G., Ugawa Y., Momose T., Tsuji S., Mannen T. (2007). Parietal dysgraphia: Characterization of abnormal writing stroke sequences, character formation and character recall. Behav. Neurol..

[5] Vandenborre D., Visch-Brink E., van Dun K., Verhoeven J., Mariën P. (2018). Oral and written picture description in individuals with aphasia. Int. J. Lang. Commun. Disord..

[6] Planton S., Jucla M., Roux F.-E., Démonet J.-F. (2013). The “handwriting brain”: A meta-analysis of neuroimaging studies of motor versus orthographic processes. Cortex.

[7] Ellis A.W. (1982). Spelling and writing (and reading and speaking). Normality and Pathology in Cognitive Functions.

[8] Patterson K. (1986). Lexical but nonsemantic spelling?. Cogn. Neuropsychol..

[9] Rapp B., Caramazza A. (2002). Selective difficulties with spoken nouns and written verbs: A single case study. J. Neurolinguistics.

[10] Caramazza A., Miceli G., Villa G., Romani C. (1987). The role of the graphemic buffer in spelling: Evidence from a case of acquired dysgraphia. Cognition.

[11] Shallice T. (1981). Neurological impairment of cognitive processes. Br. Med. Bull..

[12] Graham N.L. (2014). Dysgraphia in primary progressive aphasia: Characterisation of impairments and therapy options. Aphasiology.

[13] Hillis A.E. (2008). Cognitive processes underlying reading and writing and their neural substrates. Handb. Clin. Neurol..

[14] Mesulam M.M., Coventry C., Bigio E.H., Geula C., Thompson C., Bonakdarpour B., Gefen T., Rogalski E.J., Weintraub S. (2021). Nosology of primary progressive aphasia and the neuropathology of language. Frontotemporal Dementias: Emerging Milestones of the 21st Century.

[15] Gorno-Tempini M.L., Hillis A.E., Weintraub S., Kertesz A., Mendez M., Cappa S.F., Ogar J.M., Rohrer J.D., Black S., Boeve B.F. (2011). Classification of primary progressive aphasia and its variants. Neurology.

[16] Graham N.L. (2000). Dysgraphia in dementia. Neurocase.

[17] Graham N.L., Patterson K., Hodges J.R. (2004). When more yields less: Speaking and writing deficits in nonfluent progressive aphasia. Neurocase.

[18] Fan J.M., Gorno-Tempini M.L., Dronkers N.F., Miller B.L., Berger M.S., Chang E.F. (2020). Data-Driven, visual framework for the characterization of aphasias across stroke, post-resective, and neurodegenerative disorders over time. Front. Neurol..

[19] Sajjadi S.A., Patterson K., Nestor P.J. (2014). Logopenic, mixed, or Alzheimer-related aphasia?. Neurology.

[20] Mazzeo S., Polito C., Padiglioni S., Berti V., Bagnoli S., Lombardi G., Piaceri I., Carraro M., De Cristofaro M.T., Passeri A. (2020). Linguistic profiles, brain metabolic patterns and rates of amyloid-β biomarker positivity in patients with mixed primary progressive aphasia. Neurobiol. Aging.

[21] Utianski R.L., Botha H., Martin P.R., Schwarz C.G., Duffy J.R., Clark H.M., Machulda M.M., Butts A.M., Lowe V.J., Jack C.R. (2019). Clinical and neuroimaging characteristics of clinically unclassifiable primary progressive aphasia. Brain Lang..

[22] Henry M.L., Grasso S.M. (2018). Assessment of individuals with primary progressive aphasia. Seminars in Speech and Language.

[23] Neophytou K., Themistocleous C., Wiley R., Tsapkini K., Rapp B. Understanding and classifying the different variants of Primary Progressive Aphasia based on spelling performance. Proceedings of the Academy of Aphasia 56th Annual Meeting.

[24] Tippett D.C., Surrao K., Neophytou K., Kim H., Gallegos J., Themistocleous C., Rapp B., Hillis A.E., Tsapkini K. (2025). Written picture descriptions distinguish variants of primary progressive aphasia. J. Alzheimer’s Dis..

[25] Bonin P., Méot A., Lagarrigue A., Roux S. (2015). Written object naming, spelling to dictation, and immediate copying: Different tasks, different pathways?. Q. J. Exp. Psychol..

[26] Bonin P., Méot A., Laroche B., Bugaiska A., Perret C. (2019). The impact of image characteristics on written naming in adults. Read. Writ..

[27] Whitworth A., Webster J., Howard D. (2005). Written word production. A Cognitive Neuropsychological Approach to Assessment and Intervention in Aphasia: A Clinician’s Guide.

[28] Rapcsak S.Z., Henry M.L., Teague S.L., Carnahan S.D., Beeson P.M. (2007). Do dual-route models accurately predict reading and spelling performance in individuals with acquired alexia and agraphia?. Neuropsychologia.

[29] Weekes B.S. (2006). Acquired disorders of reading and writing: Cross-script comparisons. Behav. Neurol..

[30] Weekes B.S. (2012). Acquired dyslexia and dysgraphia across scripts. Behav. Neurol..

[31] Liu J., Ota S., Kawakami N., Kanno S., Suzuki K. (2022). Dyslexia and dysgraphia of primary progressive aphasia in Chinese: A systematic review. Front. Neurol..

[32] Peters M.D.J., Marnie C., Tricco A.C., Pollock D., Munn Z., Alexander L., McInerney P., Godfrey C.M., Khalil H. (2020). Updated methodological guidance for the conduct of scoping reviews. JBI Evid. Synth..

[33] Tricco A.C., Lillie E., Zarin W., O’Brien K.K., Colquhoun H., Levac D., Moher D., Peters M.D.J., Horsley T., Weeks L. (2018). PRISMA Extension for Scoping Reviews (PRISMAScR): Checklist and Explanation. Ann. Intern. Med..

[34] Graham N.L., Patterson K., Hodges J.R. (2000). The impact of semantic memory impairment on spelling. Evidence from semantic dementia. Neuropsychologia.

[35] Henry M.L., Wilson S.M., Babiak M.C., Mandelli M.L., Beeson P.M., Miller Z.A., Gorno-Tempini M.L. (2016). Phonological processing in primary progressive aphasia. J. Cogn. Neurosci..

[36] Tee B.L., Lorinda Kwan-Chen L.Y., Chen T.F., Yan C.T., Tsoh J., Lung-Tat Chan A., Wong A., Lo R.Y., Lu C.L., Gorno-Tempini M.L. (2022). Dysgraphia phenotypes in native Chinese speakers with primary progressive aphasia. Neurology.

[37] Benedet M., Patterson K., Gomez-Pastor K., Garcia de la Rocha M.L. (2006). Non-semantic Aspects of Language in Semantic Dementia: As Normal as They’re Said to Be?. Neurocase Neural Basis Cogn..

[38] Calabria M., Jefferies E., Sala I., Morenas-Rodríguez E., Illán-Gala I., Montal V., Fortea J., Lleó A., Costa A. (2021). Multilingualism in semantic dementia: Language-dependent lexical retrieval from degraded conceptual representations. Aphasiology.

[39] Clerc M.-T., Deprez M., Leuba G., Lhermitte B., Lopez U., von Gunten A. (2015). Atypical association of semantic dementia, corticobasal syndrome, and 4R tauopathy. Neurocase.

[40] Lavoie M., Bier N., Laforce R., Macoir J. (2020). Improvement in functional vocabulary and generalization to conversation following a self-administered treatment using a smart tablet in primary progressive aphasia. Neuropsychol. Rehabil..

[41] Grasso S.M., Shuster K.M., Henry M.L. (2017). Comparing the effects of clinicians and caregiver-admnistered lexical retrieval training for progressive anomia. Neuropsychol. Rehabil..

[42] Nickels K., Beeson P.M., Rising K., Jebahi F., Kielar A. (2023). Positive changes to written language following phonological treatment in logopenic variant primary progressive aphasia: Case report. Front. Hum. Neurosci..

[43] Rohrer J.D., Crutch S.J., Warrington E.K., Warren J.D. (2010). Progranulin-associated primary progressive aphasia: A distinct phenotype?. Neuropsychologia.

[44] Silveri M.C., Pravatà E., Brita A.C., Improta E., Ciccarelli N., Rossi P., Colosimo C. (2014). Primary progressive aphasia: Linguistic patterns and clinical variants. Brain Lang..

[45] Harris J.M., Saxon J.A., Jones M., Snowden J.S., Thompson J.C. (2019). Neuropsychological differentiation of progressive aphasic disorders. J. Neuropsychol..

[46] Code C., Ball M., Tree J., Dawe K. (2013). The effects of the initation, termination and inhibition impairments on speech rate in a case of progressive nonfluent aphasia with progressive apraxia of speech with frontotemporal degeneration. J. Neurolinguistics.

[47] Schneider S.L., Thompson C.K., Luring B. (2007). Effects of verbal plus gestural matrix training on sentence production in a patient with primary progressive aphasia. Aphasiology.

[48] Lo Monaco M.R.L., Di Tella S., Anzuino I., Ciccarelli N., Silveri M.C. (2022). Writing errors in primary progressive aphasia. Appl. Neuropsychol. Adult.

[49] Rofes A., De Aguiar V., Ficek B., Wendt H., Webster K., Tsapkini K. (2019). The role of word properties in performance on fluency tasks in people with primary progressive aphasia. J. Alzheimer’s Dis..

[50] Shim H., Hurley R.S., Rogalski E., Mesulam M.-M. (2012). Anatomic, clinical, and neuropsychological correlates of spelling errors in primary progressive aphasia. Neuropsychologia.

[51] Sepelyak K., Crinion J., Molitoris J., Epstein-Peterson Z., Bann M., Davis C., Newhart M., Heidler-Gary J., Tsapkini K., Hillis A.E. (2011). Patterns of breakdown in spelling in primary progressive aphasia. Cortex.

[52] Macoir J., Bernier J. (2002). Is surface dysgraphia tied to semantic impairment? Evidence from a case of semantic dementia. Brain Cogn..

[53] Morello García F., Difalcis M., Leiva S., Allegri R.F., Ferreres A.R. (2021). Acquired surface dysgraphia and dyslexia in the semantic variant of primary progressive aphasia: A single-case study in Spanish. Aphasiology.

[54] Teichmann M., Sanches C., Moreau J., Ferrieux S., Nogues M., Dubois B., Cacouault M., Sharifzadeh S. (2019). Does surface dyslexia/dysgraphia relate to semantic deficits in the semantic variant of primary progressive aphasia?. Neuropsychologia.

[55] Tree J.J., Kay J., Perfect T.J. (2005). “Deep” language disorders in nonfluent progressive Aphasia: An evaluation of the “summation” account of semantic errors across language production tasks. Cogn. Neuropsychol..

[56] Fushimi T., Komori K., Ikeda M., Patterson K., Ijuin M., Tanabe H. (2003). Surface dyslexia in a Japanese patient with semantic dementia: Evidence for similarity-based orthography-to-phonology translation. Neuropsychologia.

[57] Pineault J., Jolicœur P., Grimault S., Lacombe J., Brambati S.M., Bier N., Chayer C., Joubert S. (2019). A MEG study of the neural substrates of semantic processing in semantic variant primary progressive aphasia. Neurocase.

[58] Macoir J., Martel-Sauvageau V., Bouvier L., Laforce R., Monetta L. (2021). Heterogeneity of repetition abilities in logopenic variant primary progressive aphasia. Dement. Neuropsychol..

[59] Meyer A.M., Snider S.F., Eckmann C.B., Friedman R.B. (2015). Prophylactic treatments for anomia in the logopenic variant of primary progressive aphasia: Cross-language transfer. Aphasiology.

[60] Patterson K., Ralph M.A.L., Jefferies E., Woollams A., Jones R., Hodges J.R., Rogers T.T. (2006). “Presemantic” cognition in semantic dementia: Six deficits in search of an explanation. J. Cogn. Neurosci..

[61] Henry M.L., Beeson P.M., Alexander G.E., Rapcsak S.Z. (2012). Written language Impairments in Primary Progressive Aphasia: A Reflection of Damage to Central Semantic and Phonological Process. J. Cogn. Neurosci..

[62] Rumiati R.I., Foroni F., Pergola G., Rossi P., Silveri M.C. (2016). Lexical-semantic deficits in processing food and non-food items. Brain Cogn..

[63] Henry M., Rising K., DeMarco A., Miller B., Gorno-Tempini M., Beeson P. (2013). Examining the value of lexical retrieval treatment in primary progressive aphasia: Two positive cases. Brain Lang..

[64] Krajenbrink T., Croot K., Taylor-Rubin C., Nickels L. (2020). Treatment for spoken and written word retrieval in the semantic variant of primary progressive aphasia. Neuropsychol. Rehabil..

[65] Hameister I., Nickels L., Abel S., Croot K. (2017). “Do you have mowing the lawn?”—Improvements in word retrieval and grammar following constraint-induced language therapy in primary progressive aphasia. Aphasiology.

[66] Caño A., Hernández M., Ivanova I., Juncadella M., Gascón-Bayarri J., Reñé R., Costa A. (2010). When one can write SALTO as noun but not as verb: A grammatical category-specific, modality-specific deficit. Brain Lang..

[67] Hernandez M., Cano A., Costa A., Sebastiangalles N., Juncadella M., Gasconbayarri J. (2008). Grammatical category-specific deficits in bilingual aphasia. Brain Lang..

[68] Hillis A.E., Tuffiash E., Caramazza A. (2002). Modality-Specific Deterioration in Naming Verbs in Nonfluent Primary Progressive Aphasia. J. Cogn. Neurosci..

[69] Code C., Muller N., Tree J., Ball M. (2006). Syntactic impairments can emerge later: Progressive agrammatic agraphia and syntactic comprehension impairment. Aphasiology.

[70] Josephy-Hernandez S., Rezaii N., Jones A., Loyer E., Hochberg D., Quimby M., Wong B., Dickerson B.C. (2023). Automated analysis of written language in the three variants of primary progressive aphasia. Brain Commun..

[71] Taylor-Rubin C., Nickels L., Croot K. (2022). Exploring the effects of verb and noun treatment on verb phrase production in primary progressive aphasia: A series of single case experimental design studies. Neuropsychol. Rehabil..

[72] Tetzloff K.A., Duffy J.R., Clark H.M., Utianski R.L., Strand E.A., Machulda M.M., Botha H., Martin P.R., Schwarz C.G., Senjem M.L. (2019). Progressive agrammatic aphasia without apraxia of speech as a distinct syndrome. Brain.

[73] Tzoutio-Mazoyera N., Landeau B., Papathanassiou D., Crivello F., Etard O., Delcroix N., Tzourio-Mazoyer B., Joliot M. (2002). Automated Anatomical Labeling of Activations in SPM using a Macroscopic Anatomical Parcellation of the MNI MRI Single-Subject Brain. NeuroImage.

[74] Visser M., Jefferies E., Embleton K.V., Ralph M.A.L. (2012). Both the Middle Temporal Gyrus and the Ventral Anterior Temporal Area Are Crucial for Multimodal Semantic Processing: Distortion-corrected fMRI Evidence for a Double Gradient of Information Convergence in the Temporal Lobes. J. Cogn. Neurosci..

[75] Boschi V., Catricalà E., Consonni M., Chesi C., Moro A., Cappa S.F. (2017). Connected speech in Neurodegenerative Language Disorders: A Review. Front. Psychol..

[76] Mesulam M.M., Rader B.M., Sridhar J., Nelson M.J., Hyun J., Rademaker A., Geula C., Bigio E.H., Thompson C.K., Rogalski E.J. (2019). Word comprehension in temporal cortex and Wernicke area: A PPA perspective. Neurology.

[77] Wilson S.M., Henry M.L., Besbris M., Ogar J.M., Dronkers N.F., Jarrold W., Miller B.L., Gorno-Tempini M.L. (2010). Connected speech production in three variants of primary progressive aphasia. Brain.

[78] Ota S., Suzuki M., Takasaki A., Kawakami N., Morihara K., Kakinuma K., Matsubara S., Katsuse K., Iseki C., Kanno S. (2025). Dysgraphia in Japanese patients with primary progressive aphasia. Brain Lang..

[79] Ash S., Moore P., Antani S., McCawley G., Work M., Grossman M. (2006). Trying to tell a tale: Discourse impairments in progressive aphasia and frontotemporal dementia. Neurology.

[80] Ash S., Grossman M. (2015). Why study connected speech production. Cognitive Neuroscience of Natural Language Use.

[81] Ulugut H., Stek S., Wagemans L.E.E., Jutten R.J., Keulen M.A., Bouwman F.H., Prins N.D., Lemstra A.W., Krudop W., Teunissen C.E. (2022). The natural history of primary progressive aphasia: Beyond aphasia. J. Neurol..

[82] Santi G.C., Conca F., Esposito V., Polito C., Caminiti S.P., Boccalini C., Morinelli C., Berti V., Mazzeo S., Bessi V. (2024). Heterogeneity and overlap in the continuum of linguistic profile of logopenic and semantic variants of primary progressive aphasia: A Profile Analysis based on Multidimensional Scaling study. Alzheimer’s Res. Ther..

[83] Knibb J.A., Woollams A.M., Hodges J.R., Patterson K. (2009). Making sense of progressive non-fluent aphasia: An analysis of conversational speech. Brain.

[84] Meteyard L., Patterson K. (2009). The relation between content and structure in language production: An analysis of speech errors in semantic dementia. Brain Lang..

[85] Sajjadi S.A., Patterson K., Tomek M., Nestor P.J. (2012). Abnormalities of connected speech in the non-semantic variants of primary progressive aphasia. Aphasiology.

[86] Catricalà E., Polito C., Presotto L., Esposito V., Sala A., Conca F., Gasparri C., Berti V., Filippi M., Perani D. (2020). Neural correlates of naming errors across different neurodegenerative diseases: An FDG-PET study. Neurology.

[87] Conca F., Esposito V., Giusto G., Cappa S.F., Catricalà E. (2022). Characterization of the logopenic variant of primary progressive aphasia: A systematic review and meta-analysis. Ageing Res. Rev..

[88] Rozzini L., Bianchetti A., Lussignoli G., Cappa S., Trabucchi M. (1997). Surface dyslexia in an Italian patient with semantic dementia. Neurocase.

[89] Masterson J., Coltheart M., Meara P. (2017). Surface dyslexia in a language without irregularly spelled words. Surface Dyslexia.

[90] Licciardo D., Isella V., Canu E., Forestiero M., Castelnovo V., Valsecchi S., Agosta F., Filippi M., Appollonio I., Nestor P.J. (2025). Resolving the problem of surface dyslexia in Italian through inflection of irregular verbs. J. Neuropsychol..

[91] Blasi D.E., Henrich J., Adamou E., Kemmerer D., Majid A. (2022). Over-reliance on English hinders cognitive science. Trends Cogn. Sci..

[92] Grossman M., Mickanin J., Onishi K., Hughes E., D’Esposito M., Ding X.-S., Alavi A., Reivich M. (1996). Progressive nonfluent aphasia: Language, cognitive, and PET measures contrasted with probable Alzheimer’s disease. J. Cogn. Neurosci..

[93] Heitkamp N., Schumacher R., Croot K., de Langen E.G., Monsch A.U., Baumann T., Danek A. (2016). A longitudinal linguistic analysis of written text production in a case of semantic variant primary progressive aphasia. J. Neurolinguistics.

[94] Hwang Y.T., Strikwerda-Brown C., El-Omar H., Ramanan S., Hodges J.R., Burrell J.R., Piguet O., Irish M. (2021). “More than words”—Longitudinal linguistic changes in the works of a writer diagnosed with semantic dementia. Neurocase.

[95] Meyer A.M., Getz H.R., Brennan D.M., Hu T.M., Friedman R.B. (2016). Telerehabilitation of anomia in primary progressive aphasia. Aphasiology.

[96] Shah-Basak P., Fernandez A., Armstrong S.E., Hodzic-Santor B.H., Lavoie M., Jokel R., Meltzer J.A. (2022). Behavioural and neurophysiological responses to written naming treatment and high definition tDCS: A case study in advanced primary progressive aphasia. Aphasiology.

[97] Sitek E.J., Barczak A., Kluj-Kozłowska K., Kozłowski M., Barcikowska M., Sławek J. (2015). Is descriptive writing useful in the differential diagnosis of logopenic variant of primary progressive aphasia, Alzheimer’s disease and mild cognitive impairment?. Neurol. Neurochir. Polska.

[98] Sitek E.J., Barczak A., Kluj-Kozłowska K., Kozłowski M., Narożańska E., Konkel A., Dąbrowska M., Barcikowska M., Sławek J. (2015). Writing in Richardson variant of progressive supranuclear palsy in comparison to progressive non-fluent aphasia. Neurol. Neurochir. Polska.

