# Supplementary material for: Writing Abilities in Primary Progressive Aphasia: A Scoping Literature Review
Source: Brain Sci. 2026 Apr 17;16(4):420. doi: 10.3390/brainsci16040420 (PMC13114305; doi:10.3390/brainsci16040420)
Supplement: Supplementary file 1 [file brainsci-16-00420-s001.zip › Supplementary Materials_final.pdf]

Supplementary Materials

1.1. Behavioral results

1.1.1. Copying

Only one paper presented a copying task, involving Japanese characters and comparing svPPA with HC (Sakurai et al., 2021). Subjects with svPPA were impaired in copying both Kanji and Kana characters and were more accurate for frequent, familiar, and structurally simple characters in Kanji.

1.1.2. Writing to dictation

1.1.2.1. Real words

Twenty-three papers reported the *overall performance* on real words, not distinguishing between different types of word stimuli such as regular and irregular ones. See Figure 1 for the main results.

All PPA vs HC. One paper compared PPA patients (independently of their variant) with HC, reporting a worse performance for PPA (Rumiati et al., 2016).

svPPA vs HC. Nine papers compared svPPA with HC, specifically 5 were group studies (Graham et al., 2000; Henry et al., 2016; Harris et al., 2018; Silveri et al., 2014; Tee et al., 2022) and 4 reported five single cases (Benedet et al., 2006; Calabria et al., 2021; Clerc et al., 2013; Lavoie et al., 2019). In the majority of studies, including 3 group studies and 4 single cases, svPPA performed worse than HC (Graham et al., 2000; Henry et al., 2016; Tee et al., 2002; Benedet et al., 2006; Calabria et al., 2021; Clerc et al., 2013; Lavoie et al., 2019). A similar performance between svPPA and HC has been reported only in 2 group studies (Harris et al., 2018; Silveri et al., 2014) and in one single case (Calabria et al., 2021).

lvPPA vs HC. Eight papers compared lvPPA with HC, specifically 4 were group studies (Harris et al., 2018; Henry et al., 2016; Silveri et al., 2014; Tee et al., 2022) and 4 reported six single cases (Grasso et al., 2017; Lavoie et al., 2019; Nickels et al., 2023; Roher et al., 2010). In the majority of studies, including 3 group studies and 5 single cases, lvPPA showed an impaired performance compared to HC (Grasso et al., 2017; Henry et al., 2016; Lavoie et al., 2019; Nickels et al., 2023; Roher et al., 2010; Silveri et al., 2014; Tee et al., 2022). A similar performance between lvPPA and HC has been reported in the other two studies (Harris et al., 2018; Lavoie et al., 2019).

nfvPPA vs HC. Eight papers compared nfvPPA with HC, specifically 6 were group studies (Graham et al., 2004; Harris et al., 2018; Henry et al., 2016; Silveri et al., 2014; Tee et al., 2022; Thompson et al., 2007), and 2 reported two single cases (Code et al., 2013; Tree et al., 2005). No differences were reported in the majority of cases, including four group studies and one single case (Code et al., 2013; Henry et al., 2016; Harris et al., 2018, Silveri et al., 2014; Thompson et al., 2007), while the remaining studies, including two group studies and one single case, reported an impairment for nfvPPA (Graham et al., 2004; Tree et al., 2005; Tee et al., 2022).

Comparing PPA variants. Six group studies compared the three PPA variants (Silveri et al., 2014; Lo Monaco et al., 2020; Tee et al., 2022; Rofes et al., 2019; Harris et al., 2018; Ota et al., 2025). svPPA performed as nfvPPA in all studies, except in the one in Japanese (Ota et al., 2025), with svPPA performing worse than nfvPPA in Kanji and better in Kana characters. lvPPA obtained the same performance as svPPA and nfvPPA in five studies, while one study reported a worse performance in comparison to both variants (Silveri et al., 2014), and one other study showed a worse performance in comparison to nfvPPA in Kanji characters (Ota et al., 2025).

Longitudinal assessment. Five studies reported the performance of PPA over different time intervals. **svPPA.** Three papers reported svPPA performance over time, one was a group study (Graham et al., 2000) and two presented 1 single case each (Benedet et al., 2006; Clerc et al., 2015). Time points of assessment ranged from 7 to 64 months since the baseline, with either 2 (Benedet et al., 2006) or 3 assessments (Clerc et al., 2015; Graham et al., 2000). Two studies reported a progressive impairment of svPPA performance between the first and the second follow-up (Graham et al., 2000; Clerc et al., 2015), while the other study did not report a significant difference between the two time points (Benedet et al., 2006). **nfvPPA.** Two papers observed the performance of two nfvPPA cases over time (Code et al., 2006; Shah-Basak et al., 2021). Although both studies considered 3 assessments, the time elapsed between follow-ups was differed. The first and the last assessments were spaced two years in Code et al. (2006), and five years in Shah-Basak et al. (2021). A decline was reported only in Shan-Basak et al. (2021), comparing the baseline with the third assessment.

PPA vs other conditions. Two studies compared the three PPA variants with other conditions, namely with CBD (Lo Monaco et al., 2020) and with AD patients (Harris et al., 2018). In both studies, PPA had the same performance as the other conditions. One study compared the three PPA variants with AOS patients (Silveri et al., 2014). svPPA and nfvPPA showed a similar performance compared to AOS, while lvPPA had lower performance.

Qualitative scoring. Qualitative differences in errors were reported in five papers, which differed in the type and the number of errors considered, ranging from 2 in Graham et al. (2000) to 12 in

Tee at al. (2022). In addition to the different level of detail in the qualitative scoring of each study, differences were also due to the intrinsic characteristics of the specific languages (e.g., considering neographism only in Chinese). The comparison across studies was thus difficult. **svPPA.** Six studies reported a qualitative error scoring in svPPA (Graham et al., 2000; Lo Monaco et al., 2020; Sepalyak et al., 2011; Shim et al., 2012; Tee et al., 2022; Ota et al., 2025), all showing phonologically plausible errors (PPEs), defined in the Chinese study as phonologically alike writing errors, and homophone paragraphia (Tee et al., 2002). Non-phonologically plausible (NonPPEs) were less frequently reported (Graham et al., 2000; Lo Monaco et al., 2020). **lvPPA.** Four studies reported a qualitative error scoring in lvPPA (Sepalyak et al., 2011; Shim et al., 2012; Tee et al., 2022; Ota et al., 2025). Two studies reported a similar number of PPEs and NonPPEs (Shim et al., 2012; Sepalyak et al., 2011). The Chinese study reported visual and stroke dysgraphia, with orthographically similar writing errors (Tee et al., 2022), while the Japanese study showed the presence of non-response errors (Ota et al., 2025). **nfvPPA.** Five studies reported a qualitative error scoring in nfvPPA (Lo Monaco et al., 2020; Sepalyak et al., 2011; Shim et al., 2012; Tee et al., 2022; Ota et al., 2025). Differences emerged between languages. The two English studies reported a higher production of NonPPEs errors than PPEs (Shim et al., 2012; Sepalyak et al., 2011), the Italian one a similar number of PPEs, NonPPEs, and visual errors (Lo Monaco et al., 2020), the Chinese study reported graphemic paragraphias, defined as radical dysgraphia and compound word dysgraphia (Tee et al., 2022), and the Japanese study reported character omissions and stroke composition errors (Ota et al., 2025).

Effects of variables. The presence of specific effects was reported in 8 studies, which differed in the number of variables considered, ranging from 1 in Morello-Garcia et al. (2020) to 3 (i.e., Sepalyak et al., 2011), see Table Results in the Supplementary Materials. **svPPA.** Four studies assessed the presence of frequency effects in svPPA (Graham et al., 2000; Macoir et al., 2002; Morello-Garcia et al., 2020; Tee et al., 2022), all revealed a greater effect for svPPA than for HC, with patients spelling more accurately middle/high and high-frequency than low and middle/low-frequency words. Word length and concreteness were investigated in one study, which did not report any significant effect (Sepalyak et al., 2011). **lvPPA.** Only two studies considered psycholinguistic variables in lvPPA, differing in the factors and language considered. The effect of word length and concreteness was investigated in an English study, suggesting no significant impact (Sepalyak et al., 2011). A Chinese study revealed a worsening performance with the increase of homophone density and stroke numbers in lvPPA (Tee et al., 2022). **nfvPPA.** Five studies assessed the effect of stimuli's characteristics in nfvPPA (Code et al., 2013; Graham et al., 2004; Sepalyak et al., 2011; Tree et al., 2005; Tee et al., 2022). Two single cases studies

considered imageability (Code et al., 2013; Tree et al., 2005), using the same test (PALPA), reporting contrasting results, i.e., Code et al. (2013) reported no effect, while Tree et al. (2005) reported a greater impairment for low than high imageable words. The different severity of disease and of language impairment of the two patients may account for this discrepancy. The patient described in Code et al. (2013) had a higher MMSE score, and a ceiling performance in the writing to dictation task. In addition, the same patient did not show any effect of frequency, which was instead reported in a group study (Tee et al., 2022) using a different test. No effect of word length was reported in two out of the three studies examining it (Code et al., 2013; Tree et al., 2005). One study reported mixed results, specifically one nfvPPA single cases showed length effect, while the other did not (Sepalyak et al., 2011). One study assessed the effect of concreteness and did not report any differences between abstract and concrete items (Sepalyak et al., 2011).

1.1.2.2. Regular, irregular words

Fourteen studies reported the performance of writing regular and/or irregular words in PPA patients. See Figure 1 for the main results.

All PPA vs HC. One group study compared all PPA with HC, reporting a similar performance on regular words, and a worse performance on irregular words in PPA (Henry et al., 2012).

svPPA vs HC. Six studies compared svPPA with HC, 2 were group studies (Patterson et al., 2006; Teichman et al, 2019), and 4 included one single case each (Fushimi et al., 2003; Macoir et al., 2002; Morello-Garcia et al., 2020; Pineault et al., 2019). All studies showed a worse performance of svPPA in comparison to HC on irregular words (Macoir et al., 2002; Teichman et al., 2019; Pinault et al., 2019; Patterson et al., 2006; Morello-Garcia et al., 2020; Fushimi et al., 2003), while most of the studies (n = 5/6) reported a similar performance between svPPA and HC on regular words.

lvPPA vs HC. Two case series studies compared a total of 5 lvPPA patients with HC (Macoir et al., 2021; Roher et al., 2010), showing a worse performance of lvPPA in comparison to HC on both regular and irregular words.

nfvPPA vs HC. The only study conducted reported a lower performance of a group of nfvPPA than HC on irregular words (Graham et al., 2004).

Comparison between PPA variants. Two studies compared the performance among PPA variants, reporting a comparable performance for regular words among PPA variants, and a lower performance of svPPA than lvPPA and nfvPPA for irregular words (Meyer et al., 2016; Shim et al., 2012).

Effects of variables. The presence of specific variable effects was reported in 5 studies. **PPA.** One study investigated and reported a regularity effect in PPA, with irregular words spelled less accurately than regular words (Henry et al., 2012). **svPPA.** Four studies investigated and reported a regularity effect in svPPA patients (Macoir et al., 2002; Morello-Garcia et al., 2020; Teichman et al., 2021). An interaction between regularity and frequency was investigated only in Patterson et al. (2006), reporting a worse performance on irregular low-frequency words, a pattern particularly evident in those patients with a more severe semantic impairment. **lvPPA.** One study investigated the effect of regularity (Sepalyak et al., 2011): no differences were reported in the majority of the assessed single-cases. **nfvPPA.** Two studies investigated the effect of regularity (Graham et al., 2004; Sepalyak et al., 2011), with irregular words spelled less accurately than regular words in one study (Graham et al., 2004).

1.1.2.3. Non-words

Eighteen papers reported the overall performance on non-words. See Figure 1 for the main results.

All PPA vs HC. Three group studies compared PPA patients with HC (Henry et al., 2012; Rumiati et al., 2016; Shim et al., 2012). Two studies reported a comparable performance of PPA in comparison to HC (Henry et al., 2012; Rumiati et al., 2016), while the other reported an impaired performance of PPA (Shim et al., 2012).

svPPA vs HC. Nine papers compared svPPA with HC, 3 were group studies (Henry et al., 2016; Silveri et al., 2014; Teichman et al., 2021) and 6 case reports including a total of 14 single cases (Graham et al., 2000; Henry et al., 2013; Krajenbrink et al., 2020; Lavoie et al., 2019; Morello-Garcia et al., 2020; Pineault et al., 2019). In two group studies and 12 single cases a comparable performance between svPPA and HC was reported. The remaining studies (1 group study and 2 single cases) showed a worse performance of svPPA in comparison to HC.

lvPPA vs HC. Seven papers compared lvPPA with HC, 2 were group studies (Henry et al., 2016; Silveri et al., 2014), and 5 included a total of 10 single cases (Grasso et al., 2007; Henry et al., 2013; Lavoie et al., 2019; Macoir et al., 2021; Nickels et al., 2023). In the two group studies and in 6 single cases, lvPPA showed an impaired performance compared to HC (Silveri et al., 2014; Henry et al., 2016; Henry et al., 2013; Lavoie et al., 2019; Macoir et al., 2021; Nickels et al., 2023), while the remaining 4 single cases had a similar performance compared to HC (Grasso et al., 2017; Henry et al., 2013; Lavoie et al., 2019; Macoir et al., 2021).

nfvPPA vs HC. Three papers compared nfvPPA with HC, namely 2 group studies (Henry et al., 2016; Silveri et al., 2014) and 1 single case report (Tree et al., 2005). The two group studies did

not find any difference between nfvPPA and HC (Henry et al., 2016; Silveri et al., 2014), while the single case study reported an impairment for nfvPPA (Tree et al., 2005).

Comparison between PPA variants. Five papers compared the three PPA variants (Henry et al., 2016; Lo Monaco et al., 2020; Rofes et al., 2019; Shim et al., 2012; Silveri et al., 2014). In four studies nfvPPA showed a worse performance compared to svPPA, while in the remaining one the two variants did not differ (Henry et al., 2016). The comparison between nfvPPA and lvPPA showed heterogeneous results, with 1 study showing a similar performance (Shim et al., 2012), 2 studies a lower performance for lvPPA (Henry et al., 2016; Silveri et al., 2014) and 1 the opposite pattern (Rofes et al., 2019). When compared with svPPA, lvPPA had a lower performance in 3 studies, while the remaining two studies did not find any significant difference (Shim et al., 2012; Rofes et al., 2019).

Longitudinal assessment. Only one study investigated the performance of nfvPPA for 12 months including two assessments (Code et al., 2006). No difference was reported.

Comparison between PPA and other conditions. One study compared the three PPA variants with CBD patients (Lo Monaco et al., 2020), reporting a similar performance between groups. One study compared the three PPA variants with AOS patients (Silveri et al., 2014). svPPA and nfvPPA showed a similar performance compared to AOS, while lvPPA had a worse performance.

Qualitative scoring. Error analysis was reported in one study (Lo Monaco et al., 2020). It showed a higher production of NonPPEs errors (i.e., orthographic) than visual errors in svPPA.

Effects of variables. The presence of specific effects was reported in 5 studies. **svPPA**. Three studies assessed the lexicality effect in svPPA (Henry et al., 2016; Morello-Garcia et al., 2020; Teichman et al., 2021), and reported that non-words were spelled more accurately than real words, due to a specific impairment of irregular stimuli (Henry et al., 2016; Morello Garcia et al., 2020), or regular words (regular < irregular < non-words; Teichman et al., 2021). **lvPPA.** Two studies revealed a lexicality effect in lvPPA, with non-words spelled less accurately than real words (Henry et al., 2016; Nickels et al., 2023). **nfvPPA.** Letter length and lexicality were explored in one study each (Tree et al., 2005; Henry et al., 2016, respectively), and no significant effects were reported.

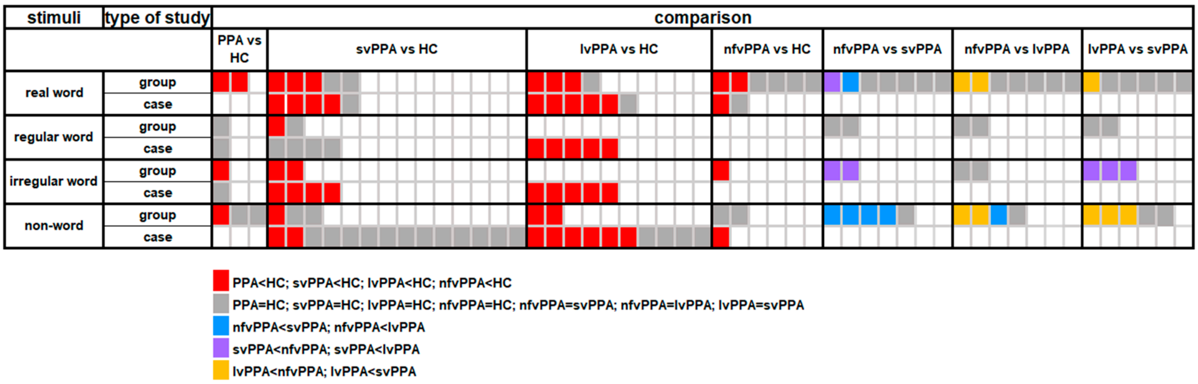

Figure S1. Summary of the results of the writing to dictation tasks, indicating when the performance was equal (=), worse (<), or better (>) in the comparisons between PPA and HC and between PPA variants. PPA = Primary Progressive Aphasia; svPPA = semantic variant of Primary Progressive Aphasia; nfvPPA = non fluent variant of Primary Progressive Aphasia; lvPPA = logopenic variant of Primary Progressive Aphasia; HC = healthy controls.

1.1.2.4 Sentences. Three studies adopted sentences as stimuli.

All PPA vs HC. One group study compared all PPA versus HC, and reported an impairment in PPA (Rumiati et al., 2016).

svPPA vs HC. Two studies compared svPPA to HC, reporting a similar performance between groups (Calabria et al., 2021; Silveri et al., 2014).

lvPPA, nfvPPA vs HC. One group study reported a lower performance of lvPPA and nfvPPA compared to HC (Silveri et al., 2014).

Comparison between PPA variants. One group study compared the performance among PPA variants (Silveri et al., 2014): lvPPA and nfvPPA performed worse than svPPA, while no significant differences emerged between nfvPPA and lvPPA.

Comparison between PPA and other conditions. One study compared the three PPA variants with AOS patients (Silveri et al., 2014). svPPA and lvPPA showed a similar performance compared to AOS, while nfvPPA showed lower performance.

1.1.2.5. Letters

svPPA vs HC. One study compared a patient with svPPA with HC, reporting a similar performance (Krajenbrink et al., 2020). A ***longitudinal assessment*** at 3 and 4 years after the baseline reported no differences. At all-time points, low-frequency letters were spelled less accurately than high-frequency ones. A regularization and multi-letter response errors (i.e., Q as CUE) were also reported in svPPA.

1.1.2.6. Numbers

All PPA vs HC. One group study compared PPA with HC, reporting an impaired performance for PPA (Rumiati et al., 2016).

1.1.3. Written naming

1.1.3.1. Overall performance

Three papers reported the overall performance on picture naming.

svPPA vs HC. One paper compared 2 patients with svPPA vs HC, reporting an impairment for svPPA patients (Lavoie et al., 2019).

lvPPA vs HC. One paper compared 3 patients with lvPPA to HC (Lavoie et al., 2019). Among the three lvPPA patients, one performed worse than controls, one performed similarly, and one could not complete the task. These differences likely reflect variability in age, disease duration, and general cognitive status. Specifically, the patient with preserved performance was younger than the one showing impairment, while the patient unable to complete the task had a longer disease duration and more widespread cognitive deficits.

nfvPPA vs HC. In two papers a total of 3 single cases reported no differences when compared to HC (Haimester et al., 2016; Thompson et al., 2007).

1.1.3.2. Objects and actions

Four papers reported the performance on written naming on objects, and 2 of them reported also the performance on actions.

All PPA vs HC. One group study compared all PPA versus HC (Rumiati et al., 2016), reporting a similar performance on objects, and a worse one on actions for PPA.

svPPA vs HC. One group study compared svPPA patients with HC by assessing objects and actions separately (Silveri et al., 2014), while two single-case studies focused only on objects (Calabria et al., 2021; Fushimi et al., 2003). On object naming, both an equal (Calabria et al., 2021) and an impaired performance (Silveri et al., 2014) was found. In action naming, svPPA patients performed poorly compared to HC (Silveri et al., 2014). In the Japanese case, including only object stimuli, performance was impaired for irregular characters but preserved for regular ones (Fushimi et al., 2003). However, the limited characterization of the stimuli - for instance, the lack of clear distinctions between regular and irregular words - hinders the interpretation of these results.

lvPPA vs HC. One group study compared lvPPA patients with HC (Silveri et al., 2014), showing comparable performance on object naming but poorer performance on action naming in the lvPPA group.

nfvPPA vs HC. Two papers compared nfvPPA with HC, considering objects (Graham et al., 2004; Silveri et al., 2014), or also actions (Silveri et al., 2014). With respect to HC, a worse performance was reported in nfvPPA in both studies for objects and actions.

Comparison between PPA variants. One paper compared the three PPA variants (Silveri et al., 2014). svPPA compared to nfvPPA performed similarly on actions, but worse on objects. LvPPA obtained a similar performance to nfvPPA on both objects and actions, while compared to svPPA, lvPPA obtained a similar performance on objects and a worse one on actions.

Comparison with other conditions. One study compared the three PPA variants with AOS and unclassifiable PPA patients (Silveri et al., 2014). svPPA and nfvPPA showed an impaired performance compared to AOS in naming of objects, while no differences were found for actions. lvPPA showed reverse pattern, with similar performance to AOS for objects and lower performance for actions. A similar performance was obtained when comparing svPPA, lvPPA, and nfvPPA with unclassifiable PPA patients, in both objects and actions.

Longitudinal assessment. Four studies reported the performance of PPA over time. **svPPA.** One paper described the longitudinal assessment of the performance of one svPPA for objects, considering two assessments overall lasting 24 months (Benedet et al., 2006). A progressive impairment over time was reported. **nfvPPA.** Three nfvPPA cases were assessed for objects and actions, over a period ranging from 4 months to 7 years (Cano et al., 2010; Hernandez et al., 2008; Hillis et al., 2002). In two of the cases, performance for both objects and actions remained stable over time, while one study reported a progressive decline specifically for actions.

Effects. The effect of word length was explored in two studies involving nfvPPA, one including only objects (Graham et al., 2004), and the other including both objects and actions (Cano et al., 2010), either showing a worse performance for longer compared to shorter words (Graham et al., 2004) or no effects (Cano et al., 2010). The role of frequency and imageability was investigated in one study (Cano et al., 2010), failing to report significant effects.

1.1.4. Generation of written sentences

svPPA vs HC. One study compared the performance of a single svPPA patient with HC (Benedet et al., 2006), showing a mild abnormal total score on lexical, thematic, and syntactic components (see Table Results in the Supplementary Materials). Qualitatively, the patient showed difficulty in

producing atypical relative clauses (direct and indirect passive constructions), with auxiliary omission, and a tendency to replace passive sentences with active ones.

lvPPA vs HC. One study considered one single lvPPA patient with progranulin mutation (Roher et al., 2010), who reported an impaired syntactic performance-based score.

Longitudinal assessment. Two studies assessed the performance of PPA over time. **svPPA**. One study compared the performance of one svPPA case at two-time points, with a follow-up at 52 months, when the patient produced a lower performance (Benedet et al., 2006). **nfvPPA**. One study assessed a nfvPPA patient for 26 months at 5 time points, however using two different writing tasks over time. The adaptation of PALPA 39 was used for the first two time points, i.e. at baseline and 10 months, while a Verb Generation Task was used in the subsequent three different time points, i.e. at 14, 18, and 26 months from baseline (Code et al., 2006). Both tasks scores considered different linguistic components, see Table Results in Supplementary Materials. At 10 months follow-up, the patient obtained a significant reduction in the total number of words and of function words, and an increase in the omissions of determiner and of inappropriate arguments. Pronouns and "I" as initial were also reduced to zero. No differences were reported using the Verb Generation Task.

1.1.5. Written description

PPA vs HC. One study compared PPA versus HC. PPA showed a worse performance in the number of units of speech (Josephy-Hernandez et al., 2023).

svPPA vs HC. Two group studies (Josephy-Hernandez et al., 2023; Tippett et al., 2025), and 3 case reports including five patients (Krajenbrink et al., 2020; Taylor-Rubin et al., 2021; Hwang et al., 2021) compared svPPA to HC. Four studies adopted a written picture description task (Josephy-Hernandez et al., 2023; Tippett et al., 2025; Krajenbrink et al., 2020; Taylor-Rubin et al., 2021), and one a spontaneous writing task (Hwand et al., 2021). The two group studies adopted the Picnic figure of the Western Aphasia Battery (Josephy-Hernandez et al., 2023) and the Boston Diagnostic Aphasia Examination Cookie Theft Picture (Tippett et al., 2025), respectively, and both reported a reduced number of Content Units (CUs) in svPPA patients. Additionally, one showed a greater Content Unit/Units of speech ratio (Josephy-Hernandez et al., 2023) and the other a greater percentage of Noun CUs, a reduced percentage of Verb Phrase CUs, Communication Efficiency, number of total Content Words, Core Lexicon Words, and an equal performance in adjective CUs, Verb CUs, preposition phrase CUs, total words, nouns per words, functions words, pronouns/total words, adjectives/total words, adverbs/total words, articles/total words, conjunction/total words and prepositions/total words (Tippett et al., 2025).

Two case reports used the CAT figure, adopting a total score, which was under cut-off in 3 patients (Krajenbrink et al., 2020; Taylor-Rubin et al., 2021). The other case report compared the characteristics of the novel written by a svPPA with those of novels produced by 20 contemporary authors (Hwang et al., 2021). Results showed that the svPPA patient produced more nouns and adverbs, with a higher lexical density, and less unique adjectives. No differences were found assessing features as familiarity, concreteness, imageability, meaningfulness, age of acquisition, co-occurence probability and semantic distinctiveness, and in the unique lemmas, unique nouns, number of verbs, unique verbs, total adjectives, unique adverbs reported.

lvPPA vs HC. Two group studies (Josephy-Hernandez et al., 2023; Tippett et al., 2025), and 2 single cases (Taylor-Rubin et al., 2021; Meyer et al., 2015) compared the performance of lvPPA with HC. The four studies used three different images and scores. A worse performance in lvPPA than in HC was reported in the number of Content Units (Josephy-Hernandez et al., 2023) and Communication Efficiency (Tippett et al., 2025), while an equal performance was found in Content Units/Units of speech ratio (Josephy-Hernandez et al., 2023) and in adjective CUs, verb CUs, preposition phrase CUs, total words, nouns per words, functions words, pronouns/total words, adjectives/total words, adverbs/total words, articles/total words, conjunction/total words and prepositions/total words (Tippett et al., 2025). The two single case studies reported a normal performance on the total score using the CAT (Taylor-Rubin et al., 2021) or the Cookie Theft picture (Meyer et al., 2015).

nfvPPA vs HC. Four group studies (Grossman et al., 1996; Graham et al., 2004; Jospehy-Hernandez et al., 2023; Tippett et al., 2025) and one single-case study (Code et al., 2006) compared nfvPPA with HC. Four adopted a written picture description task (Graham et al., 2004; Josephy-Hernandez et al., 2023; Tippett et al., 2025; Code et al. 2026) and one a spontaneous writing task (Grossman et al., 1996). Three studies reported lower performance in nfvPPA on produced units (Code et al., 2006; Graham et al., 2004; Tippett et al., 2025). Additionally, only one reported an equal performance on adjective CUs, verb CUs, preposition phrase CUs, total words, functions words, pronouns/total words, adjectives/total words, adverbs/total words, articles/total words, conjunction/total words and prepositions/total words (Tippett et al., 2025). Six lexical features were examined in at last two studies: nfvPPA compared to HC showed a reduction of the total number of words, verbs, function words, content words, and words per minute (Graham et al., 2004; Code et al., 2006; Tippett et al., 2025). The study adopted the spontaneous writing task (Grossman et al., 1996) showed a worse performance on semantic, grammatical content, and mechanical attributes. Additional features analyzed are listed in the Supplementary Materials.

Comparison between PPA variants. Two studies compared the performance of the three PPA variants. One study showed a higher content unit/units of speech ratio in nfvPPA and svPPA than lvPPA, while no differences among variants in the content units and in the total units of speech (Josephy-Hernandez et al., 2023); the other highlighted a lower performance of svPPA in comparison to lvPPA in the total number of words, total number of syllables, total number of content words, in the number of particles/total words, in the percentage of CUs, noun phrase CUs, verb phrase CUs, Core Lexicon Words, and an equal performance in adjective CUs, verb CUs, preposition phrase CUs, total words, functions words, pronouns/total words, adjectives/total words, adverbs/total words, conjunction/total words and prepositions/total words (Tippett et al., 2025)

Comparison between PPA variants and other conditions. Three studies compared PPA with other neurodegenerative conditions: nfvPPA with PSP-RS (Sitek et al., 2015a), lvPPA with MCI and AD (Sitek et al., 2015b), and nfvPPA with AD (Grossman et al., 1996). Two used a written picture description task (Sitek et al., 2015 a and b) and one a spontaneous writing task (Grossman et al., 1996). Compared to PSP-RS, nfvPPA patients produced fewer words (both nouns and verbs) and sentences, made fewer letter errors and exhibited less micrographia (Sitek et al., 2015a). In contrast, lvPPA patients, relative to those with AD, produced a higher number of sentences and verbs, as well as more graphemic paragraphias (Sitek et al., 2015b). No differences were reported in nfvPPA compared to AD patients (Grossman et al., 1996).

Longitudinal assessment. Two studies**,** one using a written picture description task (Code et al., 2006) and the other a spontaneous writing task (Heitkamp et al., 2016), reported a longitudinal assessment. A single nfvPPA case was evaluated at three time points: at baseline and after 13 months using the *Cookie Theft* figure, and after an additional 16 months using the *Picnic* scene (Code et al., 2006). Over time, the patient showed a progressive decline in grammatical accuracy, with fewer verbs, sentences, and correct inflections at the second and third assessments compared to baseline. An increase in constructions lacking verbs was observed at the second time point, while by the third, there was also a marked reduction in total speaking time, information units, words, determiners, and function words. The study adopting the spontaneous writing task analyzed two weeks' entries per year over 12 years in a single svPPA case (Heitkamp et al., 2016). An increase in the number of total words was reported, characterized by fewer newly used words and a higher production of words with high frequency. An increase of semantic paraphasia, and errors in the composition of the sentences, with inversions or repetitions, were detected, together with the inappropriate use of quotation marks and the use of simple connectives.

References

Benedet, M., Patterson, K., Gomez-Pastor, I., & Luisa Garcia de la Rocha, M. (2006). ‘Non-semantic’aspects of language in semantic dementia: As normal as they’re said to be?. Neurocase, 12(1), 15-26.

Calabria, M., Jefferies, E., Sala, I., Morenas-Rodríguez, E., Illán-Gala, I., Montal, V., ... & Costa, A. (2021). Multilingualism in semantic dementia: Language-dependent lexical retrieval from degraded conceptual representations. Aphasiology, 35(2), 240-266.

Caño, A., Hernández, M., Ivanova, I., Juncadella, M., Gascón-Bayarri, J., Reñé, R., & Costa, A. (2010). When one can write SALTO as noun but not as verb: A grammatical category-specific, modality-specific deficit. Brain and Language, 114(1), 26-42.

Clerc, M. T., Deprez, M., Leuba, G., Lhermitte, B., Lopez, U., & von Gunten, A. (2015). Atypical association of semantic dementia, corticobasal syndrome, and 4R tauopathy. Neurocase, 21(1), 1-15.

Code, C., Ball, M., Tree, J., & Dawe, K. (2013). The effects of initiation, termination and inhibition impairments on speech rate in a case of progressive nonfluent aphasia with progressive apraxia of speech with frontotemporal degeneration. Journal of Neurolinguistics, 26(6), 602-618.

Code, C., Muller, N., Tree, J., & Ball, M. (2006). Syntactic impairments can emerge later: Progressive agrammatic agraphia and syntactic comprehension impairment. Aphasiology, 20(9), 1035-1058.

Fushimi, T., Komori, K., Ikeda, M., Patterson, K., Ijuin, M., & Tanabe, H. (2003). Surface dyslexia in a Japanese patient with semantic dementia: Evidence for similarity-based orthography-to-phonology translation. Neuropsychologia, 41(12), 1644-1658.

Graham, N. L., Patterson, K., & Hodges, J. R. (2000). The impact of semantic memory impairment on spelling: Evidence from semantic dementia. Neuropsychologia, 38(2), 143-163.

Graham, N. L., Patterson, K., & Hodges, J. R. (2004). When more yields less: Speaking and writing deficits in nonfluent progressive aphasia. Neurocase, 10(2), 141-155.

Grossman, M., Mickanin, J., Onishi, K., Hughes, E., D'Esposito, M., Ding, X. S., ... & Reivich, M. (1996). Progressive nonfluent aphasia: language, cognitive, and PET measures contrasted with probable Alzheimer's disease. Journal of cognitive neuroscience, 8(2), 135-154.

Hameister, I., Nickels, L., Abel, S., & Croot, K. (2017). “Do you have mowing the lawn?”–improvements in word retrieval and grammar following constraint-induced language therapy in primary progressive aphasia. Aphasiology, 31(3), 308-331.

Harris, J. M., Saxon, J. A., Jones, M., Snowden, J. S., & Thompson, J. C. (2019).N europsychological differentiation of progressive aphasic disorders. Journal of Neuropsychology, 13(2), 214-239.

Heitkamp, N., Schumacher, R., Croot, K., de Langen, E. G., Monsch, A. U., Baumann, T., & Danek, A. (2016). A longitudinal linguistic analysis of written text production in a case of semantic variant primary progressive aphasia. Journal of Neurolinguistics, 39, 26-37.

Henry, M. L., Beeson, P. M., Alexander G. E., & Rapcsak S. Z. (2012). Written language impairments in primary progressive aphasia: a reflection of damage to central semantic and phonological processes. J Cogn Neurosci, 24 (2), 261-275.

Henry, M. L., Rising, K., DeMarco, A. T., Miller, B. L., Gorno-Tempini, M. L., & Beeson, P. M. (2013). Examining the value of lexical retrieval treatment in primary progressive aphasia: Two positive cases. Brain and language, 127(2), 145-156.

Henry, M. L., Wilson S. M., Babiak M. C., Mandelli M. L., Beeson P. M., Miller Z. A., Gorno-Tempini M.L. (2016). Phonological processing in primary progressive aphasia. J Cogn Neurosci, 28(2): 210-222.

Hernández, M., Caño, A., Costa, A., Sebastián-Gallés, N., Juncadella, M., & Gascón-Bayarri, J. (2008). Grammatical category-specific deficits in bilingual aphasia. Brain and language, 107(1), 68-80.

Hillis, A. E., Tuffiash, E., & Caramazza, A. (2002). Modality-specific deterioration in naming verbs in nonfluent primary progressive aphasia. Journal of cognitive neuroscience, 14(7), 1099-1108.

Hwang, Y. T., Strikwerda-Brown, C., El-Omar, H., Ramanan, S., Hodges, J. R., Burrell, J. R., ... & Irish, M. (2021). "More than words"–Longitudinal linguistic changes in the works of a writer diagnosed with semantic dementia. Neurocase, 27(3), 243-252.

Josephy-Hernandez, S., Rezaii, N., Jones, A., Loyer, E., Hochberg, D., Quimby, M., ... & Dickerson, B. C. (2023). Automated analysis of written language in the three variants of primary progressive aphasia. Brain Communications, 5(4), fcad202.

Krajenbrink, T., Croot, K., Taylor-Rubin, C., & Nickels, L. (2020). Treatment for spoken and written word retrieval in the semantic variant of primary progressive aphasia. Neuropsychological Rehabilitation.

Lavoie, M., Bier, N., Laforce Jr, R., & Macoir, J. (2020). Improvement in functional vocabulary and generalization to conversation following a self-administered treatment using a smart tablet in primary progressive aphasia. Neuropsychological Rehabilitation.

Lo Monaco, M. R., Di Tella, S., Anzuino, I., Ciccarelli, N., & Silveri, M. C. (2022). Writing errors in primary progressive aphasia. Applied Neuropsychology: Adult, 29(4), 802-809.

Macoir, J., & Bernier, J. (2002). Is surface dysgraphia tied to semantic impairment? Evidence from a case of semantic dementia. Brain and Cognition, 48(2-3), 452-457.

Macoir, J., Martel-Sauvageau, V., Bouvier, L., Laforce, R., & Monetta, L. (2021). Heterogeneity of repetition abilities in logopenic variant primary progressive aphasia. Dementia & Neuropsychologia, 15(03), 405-412.

Meyer, A. M., Getz, H. R., Brennan, D. M., Hu, T. M., & Friedman, R. B. (2016). Telerehabilitation of anomia in primary progressive aphasia. Aphasiology, 30(4), 483-507.

Meyer, A. M., Snider, S. F., Eckmann, C. B., & Friedman, R. B. (2015). Prophylactic treatments for anomia in the logopenic variant of primary progressive aphasia: Cross-language transfer. Aphasiology, 29(9), 1062-1081.

Morello García, F., Difalcis, M., Leiva, S., Allegri, R. F., & Ferreres, A. R. (2021). Acquired surface dysgraphia and dyslexia in the semantic variant of primary progressive aphasia: a single-case study in Spanish. Aphasiology, 35(6), 783-804.

Nickels, K., Beeson, P. M., Rising, K., Jebahi, F., & Kielar, A. (2023). Positive changes to written language following phonological treatment in logopenic variant primary progressive aphasia: Case report. Frontiers in Human Neuroscience, 16, 1006350.

Ota, S., Suzuki, M., Takasaki, A., Kawakami, N., Morihara, K., Kakinuma, K., ... & Suzuki, K. (2025). Dysgraphia in Japanese patients with primary progressive aphasia. Brain and Language, 271, 105655.

Patterson, K., Ralph, M. A. L., Jefferies, E., Woollams, A., Jones, R., Hodges, J. R., & Rogers, T. T. (2006). "Presemantic" cognition in semantic dementia: Six deficits in search of an explanation. Journal of cognitive neuroscience, 18(2), 169-183.

Pineault, J., Jolicœur, P., Grimault, S., Lacombe, J., Brambati, S. M., Bier, N., ... & Joubert, S. (2019). A MEG study of the neural substrates of semantic processing in semantic variant primary progressive aphasia. Neurocase, 25(3-4), 118-129.

Rofes, A., De Aguiar, V., Ficek, B., Wendt, H., Webster, K., & Tsapkini, K. (2019). The role of word properties in performance on fluency tasks in people with primary progressive aphasia. Journal of Alzheimer's Disease, 68(4), 1521-1534.

Rohrer, J. D., Crutch, S. J., Warrington, E. K., & Warren, J. D. (2010). Progranulin-associated primary progressive aphasia: a distinct phenotype?. Neuropsychologia, 48(1), 288-297.

Rumiati, R. I., Foroni, F., Pergola, G., Rossi, P., & Silveri, M. C. (2016). Lexical-semantic deficits in processing food and non-food items. Brain and cognition, 110, 120-130.

Sakurai, Y., Uchiyama, Y., Takeda, A., & Terao, Y. (2021). On-Reading (Chinese-style pronunciation) predominance over kun-reading (native Japanese pronunciation) in Japanese semantic dementia. Frontiers in Human Neuroscience, 15, 700181.

Sepelyak, K., Crinion, J., Molitoris, J., Epstein-Peterson, Z., Bann, M., Davis, C., ... & Hillis, A. E. (2011). Patterns of breakdown in spelling in primary progressive aphasia. Cortex, 47(3), 342-352.

Shah-Basak, P., Fernandez, A., Armstrong, S. E., Hodzic-Santor, B. H., Lavoie, M., Jokel, R., & Meltzer, J. A. (2022). Behavioural and neurophysiological responses to written naming treatment and high definition tDCS: A case study in advanced primary progressive aphasia. Aphasiology, 36(10), 1182-1205.

Shim, H., Hurley, R. S., Rogalski, E., & Mesulam, M. M. (2012). Anatomic, clinical, and neuropsychological correlates of spelling errors in primary progressive aphasia. Neuropsychologia, 50(8), 1929-1935.

Silveri, M. C., Pravatà, E., Brita, A. C., Improta, E., Ciccarelli, N., Rossi, P., & Colosimo, C. (2014). Primary progressive aphasia: Linguistic patterns and clinical variants. Brain and language, 135, 57-65.

Sitek, E. J., Barczak, A., Kluj-Kozłowska, K., Kozłowski, M., Barcikowska, M., & Sławek, J. (2015). Is descriptive writing useful in the differential diagnosis of logopenic variant of primary progressive aphasia, Alzheimer's disease and mild cognitive impairment?. Neurologia i neurochirurgia polska, 49(4), 239-244.

Sitek, E. J., Barczak, A., Kluj-Kozłowska, K., Kozłowski, M., Narożańska, E., Konkel, A., ... & Sławek, J. (2015). Writing in Richardson variant of progressive supranuclear palsy in comparison to progressive non-fluent aphasia. Neurologia i neurochirurgia polska, 49(4), 217-222.

Taylor-Rubin, C., Nickels, L., & Croot, K. (2022). Exploring the effects of verb and noun treatment on verb phrase production in primary progressive aphasia: A series of single case experimental design studies. Neuropsychological rehabilitation, 32(6), 1121-1163.

Tee, B. L., Lorinda Kwan-Chen, L. Y., Chen, T. F., Yan, C. T., Tsoh, J., Lung-Tat Chan, A., ... & Gorno-Tempini, M. L. (2022). Dysgraphia phenotypes in native Chinese speakers with primary progressive aphasia. Neurology, 98(22), e2245-e2257.

Teichmann, M., Sanches, C., Moreau, J., Ferrieux, S., Nogues, M., Dubois, B., ... & Sharifzadeh, S. (2019). Does surface dyslexia/dysgraphia relate to semantic deficits in the semantic variant of primary progressive aphasia?. Neuropsychologia, 135, 107241.

Tippett, D. C., Surrao, K., Neophytou, K., Kim, H., Gallegos, J., Themistocleous, C., ... & Tsapkini, K. (2025). Written picture descriptions distinguish variants of primary progressive aphasia. Journal of Alzheimer’s Disease, 13872877251376381.

Tree, J. J., Kay, J., & Perfect, T. J. (2005). “Deep” language disorders in nonfluent progressive Aphasia: an evaluation of the “summation” account of semantic errors across language production tasks. Cognitive neuropsychology, 22(6), 643-659.

Utianski, R. L., Botha, H., Martin, P. R., Schwarz, C. G., Duffy, J. R., Clark, H. M., ... & Josephs, K. A. (2019). Clinical and neuroimaging characteristics of clinically unclassifiable primary progressive aphasia. Brain and language, 197, 104676.
